# Structural basis for linker histone H5–nucleosome binding and chromatin fiber compaction

**DOI:** 10.1038/s41422-024-01009-z

**Published:** 2024-08-05

**Authors:** Wenyan Li, Jie Hu, Feng Song, Juan Yu, Xin Peng, Shuming Zhang, Lin Wang, Mingli Hu, Jia-Cheng Liu, Yu Wei, Xue Xiao, Yan Li, Dongyu Li, Hui Wang, Bing-Rui Zhou, Linchang Dai, Zongjun Mou, Min Zhou, Haonan Zhang, Zheng Zhou, Huidong Zhang, Yawen Bai, Jin-Qiu Zhou, Wei Li, Guohong Li, Ping Zhu

**Affiliations:** 1 Key Laboratory of Epigenetic Regulation and Intervention, Institute of Biophysics, Chinese Academy of Sciences, Beijing, China; 2 University of Chinese Academy of Sciences, Beijing, China; 3 New Cornerstone Science Laboratory, Frontier Science Center for Immunology and Metabolism, Hubei Key Laboratory of Cell Homeostasis, College of Life Sciences, TaiKang Center for Life and Medical Sciences, Wuhan University, Wuhan, Hubei China; 4 Shandong Key Laboratory of Biophysics, Institute of Biophysics, Dezhou University, Dezhou, Shangdong China; 5 Department of Public Health Laboratory Sciences, West China School of Public Health, Sichuan University, Chengdu, Sichuan China; 6 The State Key Laboratory of Molecular Biology, CAS Center for Excellence in Molecular Cell Science, Shanghai Institute of Biochemistry and Cell Biology, Chinese Academy of Sciences, Shanghai, China; 7 National Laboratory for Condensed Matter Physics and Key Laboratory of Soft Matter Physics, Institute of Physics, Chinese Academy of Sciences, Beijing, China; 8 Laboratory of Biochemistry and Molecular Biology, National Cancer Institute, National Institutes of Health, Bethesda, MD USA; 9 Research Center for Environment and Female Reproductive Health, The Eighth Affiliated Hospital, Sun Yat-sen University, Shenzhen, Guangdong China

**Keywords:** Cryoelectron microscopy, Chromatin structure

## Abstract

The hierarchical packaging of chromatin fibers plays a critical role in gene regulation. The 30-nm chromatin fibers, a central-level structure bridging nucleosomal arrays to higher-order organizations, function as the first level of transcriptional dormant chromatin. The dynamics of 30-nm chromatin fiber play a crucial role in biological processes related to DNA. Here, we report a 3.6-angstrom resolution cryogenic electron microscopy structure of H5-bound dodecanucleosome, i.e., the chromatin fiber reconstituted in the presence of linker histone H5, which shows a two-start left-handed double helical structure twisted by tetranucleosomal units. An atomic structural model of the H5-bound chromatin fiber, including an intact chromatosome, is built, which provides structural details of the full-length linker histone H5, including its N-terminal domain and an HMG-motif-like C-terminal domain. The chromatosome structure shows that H5 binds the nucleosome off-dyad through a three-contact mode in the chromatin fiber. More importantly, the H5-chromatin structure provides a fine molecular basis for the intra-tetranucleosomal and inter-tetranucleosomal interactions. In addition, we systematically validated the physiological functions and structural characteristics of the tetranucleosomal unit through a series of genetic and genomic studies in *Saccharomyces cerevisiae* and in vitro biophysical experiments. Furthermore, our structure reveals that multiple structural asymmetries of histone tails confer a polarity to the chromatin fiber. These findings provide structural and mechanistic insights into how a nucleosomal array folds into a higher-order chromatin fiber with a polarity in vitro and in vivo.

## Introduction

In eukaryotes, genomic DNA is hierarchically packaged into different levels of chromatin organization in the nucleus. Understanding the structure and dynamics of chromatin fibers, and transitions between accessible forms of nucleosomal arrays and compacted forms of chromatin fibers is important to illuminate the biological roles of chromatin in the regulation of gene expression and other DNA-dependent activities.^1^ The nucleosome is the fundamental repeating unit of chromatin.^2,3^ The structures of nucleosome core particles (NCPs) have been resolved at high resolution by X-ray crystallography.^4^ Recently, particularly with the help of cryogenic electron microscopy (cryo-EM) techniques, various structures of NCPs in complex with associated proteins, such as chromatin remodelers, modifying enzymes, and chaperons, have been resolved.^5,6^ Nucleosomes are connected by a segment of linker DNA to form 10-nm “beads-on-a-string” nucleosomal arrays, which are further organized into a more condensed 30-nm chromatin fiber by the linker histones H1/H5.^7,8^ Using the newly developed 3C (chromosome conformation capture)-based technique and high-resolution microscopic methods, the higher-order spatial organization of chromatin in the nucleus has been explored at unprecedented resolution, revealing that chromatin fibers are further configured into chromatin loops, topologically associating domains (TADs), and chromatin compartments in the nucleus.^9–11^

Over the past decades, the structures and dynamics of 30-nm chromatin fibers have been extensively studied by a variety of techniques, including analytical ultracentrifugation (AUC), X-ray crystallography, and cryo-EM.^12–15^ Based on the studies, the folding principles of the chromatin fiber are mainly categorized into two types of models, i.e., the one-start solenoid model in which nucleosomes are arranged in a single helix with a bent linker DNA^7,16^ or the two-start cross-linker model in which nucleosomes are organized in a zig-zag configuration and stacked in a double helix with a relatively straight linker DNA.^17–20^ Previously, we determined the 3-dimensional (3D) cryo-EM structures of the reconstituted chromatin fibers containing linker histone H1.4 at a resolution of 11 angstroms (Å), showing a two-start left-handed double-helical structure twisted by tetranucleosomal units with a zig-zag configuration.^21^ Subsequently, using single-molecule magnetic tweezers, we further confirmed that the tetranucleosome is a stable structural building block of the chromatin fiber in vitro and found that the histone chaperone FACT can destabilize the tetranucleosome for gene activation.^22^ These observations in vitro are supported by several recent super-resolution imaging and micro-C genomic studies showing that nucleosomes are organized into discrete “nucleosome clutches” or “tetranucleosomal folding motifs” along the chromatin fiber in both yeast and mammalian cells, respectively.^23–26^

Linker histones H1/H5 have been shown to play a fundamental role in chromatin compaction through different mechanisms.^27–29^ Abundant in higher eukaryotic cells, variant linker histone isoforms were found to adopt a tripartite structure, composed of a structured central globular domain (GD) (~80 residues) flanked by a short N-terminal domain (NTD) (20–35 residues) and a long C-terminal domain (CTD) (~100 residues).^30^ The GD plays an important role in the binding and locating of linker histones in the nucleosome, and the CTD is believed to be a major determinant of chromatin folding.^31^ As the NTD and CTD of linker histone H1/H5 are mostly disordered in solution, no structural information for the full-length linker histone is available yet. Previously, various studies suggested that the GD of linker histone binds nucleosome core either on-dyad^30,32–34^ or off-dyad.^35–37^ In our previous cryo-EM structure, the linker histone H1.4 was found to directly interact with both the dyad and the linker DNA in a three-contact binding mode, and the GD of H1.4 shows an apparent off-dyad asymmetric binding to the nucleosome core.^21^ However, several recent structural studies on the linker histone H1.0/H5-bound mononucleosome^38,39^ or 4-nucleosomal arrays^40^ revealed an on-dyad binding mode for the GD of H1/H5. Based on the structural studies, it had been suggested that the linker histones with different binding modes could compact nucleosomal arrays to form distinct higher-order chromatin structures.^38^ Nevertheless, it remains controversial as to how the linker histone H1/H5 binds the nucleosome core and how the linker histone facilitates the folding of chromatin fibers.

Moreover, our previous cryo-EM structures suggested that the tetranucleosomal units are important building blocks of the chromatin fiber, and are stabilized and bridged by nucleosome–nucleosome and H1.4–H1.4 interactions.^21^ Due to the relatively low resolution (11 Å), the structural basis of the detailed interactions between linker histones and nucleosome cores remain elusive, although the building blocks are well resolved. Furthermore, the structure of higher-order chromatin with different linker histones, such as H5, is also worthy of study. In this study, we determined the 3D cryo-EM structure of the H5-bound dodecanucleosome reconstituted with 12× 177 bp_601 DNA, core histones (H2A, H2B, H3, and H4), and linker histone H5 (termed as the H5-chromatin fiber thereafter) at 3.6 Å resolution. In the cryo-EM structure, we can observe the full-length linker histone H5 and the majority of core histone tails. Based on the EM density map, we built an atomic model of the H5-chromatin fiber. We then systematically validated the physiological functions and structural features of the tetranucleosome folding unit in budding yeast, using a series of genetic, genomic, and biophysical approaches. Moreover, we revealed multiple structural asymmetries of histone tails which confer a polarity to the H5-chromatin fiber. This work provides a fine structural basis and mechanistic insights for understanding the assembly and polarity of the chromatin fiber and its potential roles in asymmetrically reading genetic information during transcription.

## Results

### Overall structure

#### H5-bound dodecanucleosome presents a two-start double-helical structure

**Fig. 1:**
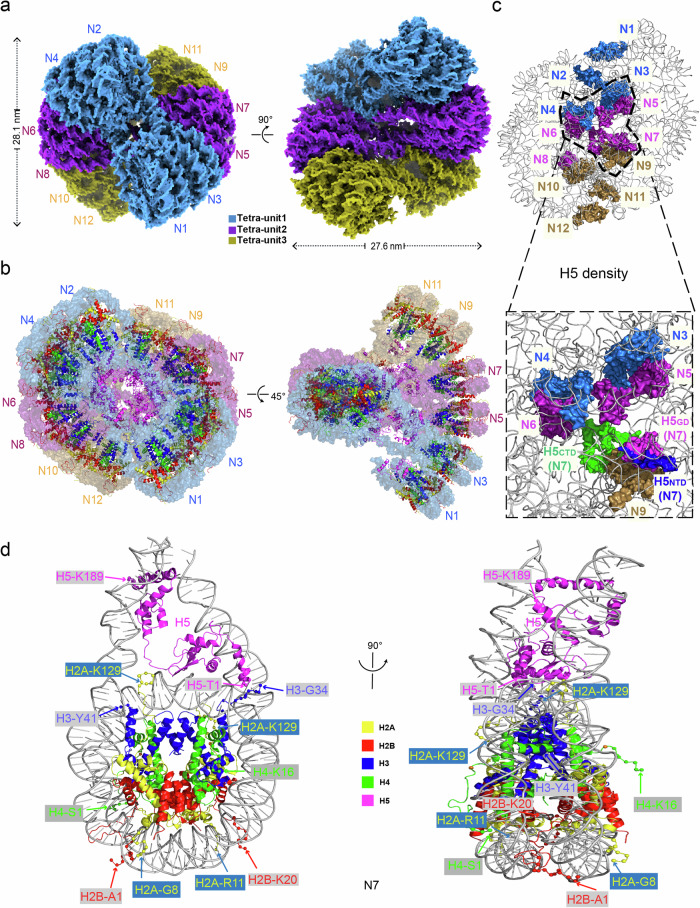
Cryo-EM structure of the chromatin fiber reconstituted with 12× 177 bp_601 DNA and linker histone H5. **a** The 3D reconstructed cryo-EM map of the H5-chromatin fiber at 3.6 Å resolution was viewed from two different angles. Three tetranucleosomal structural units are highlighted by different colors. **b** The atomic model of H5-chromatin fiber is viewed from two different angles. For viewing purposes, DNA is shown in partly transparent surface mode and colored as in **a**. Histone proteins are shown in cartoon representation with a color scheme as in **c**. **c** The dodecanucleosomal H5-chromatin fiber structure shows the location of linker histone H5 and a 1:1 stoichiometric association of H5:NCP. For clarity, only the DNA and H5 moieties are shown. The inset shows details of H5 in the dashed area. **d** Atomic structure of a representative chromatosome of H5-chromatin fiber, N7, viewed from two angles. The first and last resolved residues of each histone in N7 are labeled.

The H5-chromatin fiber, reconstituted with 12× 177 bp_601 DNA, core histones, and full-length linker histone H5 (Supplementary information, Fig. S1a, b) was prepared similarly to the H1.4-chromatin fiber as described previously.^21^ As shown in the cryo-EM micrographs, the reconstituted H5-chromatin fibers display as homogeneously compacted particles (Supplementary information, Fig. S1c), which is consistent with the AUC analysis (Supplementary information, Fig. S1d). About 43,000 particles were visually screened and semi-automatically selected for the 2D classification and the subsequent 3D reconstruction (Supplementary information, Fig. S2a–f). After rounds of 3D classification and refinements, we determined the 3D cryo-EM structure of the reconstituted H5-chromatin fiber at 3.6 Å resolution (Fig. 1a; Supplementary information, Fig. S2g and Video S1). Similar to our previous structures of the H1.4-chromatin fiber, the overall 3D architecture of the H5-chromatin fiber comprises 3 tetranucleosomal structural units twisted against each other, forming a two-start left-handed double-helical structure with a zig-zag configuration. For clarity, 3 tetranucleosomal structural units are defined as unit 1 for nucleosomes N1 to N4, unit 2 for N5 to N8, and unit 3 for N9 to N12, respectively. Importantly, based on the EM density map at the near-atomic resolution (3.6 Å) (Supplementary information, Fig. S2d), we built the atomic model of the reconstituted H5-chromatin fiber (Fig. 1b). In the cryo-EM reconstructed density, not only the nucleosomes but also the linker histones (H5), can be located unambiguously according to the EM density map (Fig. 1c; Supplementary information, Video S2). Notably, most of the core histone tails, including almost all of the H2A, H2B, H4, and the majority of H3 tails (the furthest H3-N tails can be traced up to A21) can be identified and modeled from different well-resolved nucleosomes of the H5-chromatin fiber, e.g., N7 (Fig. 1d; Supplementary information, Video S3), although not all of the histone tails are shown in the same nucleosome. Moreover, in each chromatosome, some distinguishable extra densities, which are located between the linker DNA of two adjacent nucleosomes and in close contact with the N-terminal end of H5, are readily visible (Supplementary information, Fig. S3a). These densities can accommodate ~6–11 amino acids and are likely contributed by one of the far-end N-tails of H3 in the nucleosome, e.g., H3 1–18, given their relative locations to the resolved H3 densities, e.g., about 12 Å in distance from the resolved H3 residue Thr22 in N9 (Supplementary information, Fig. S3b–d).

#### The atomic structure of full-length linker histone H5

**Fig. 2:**
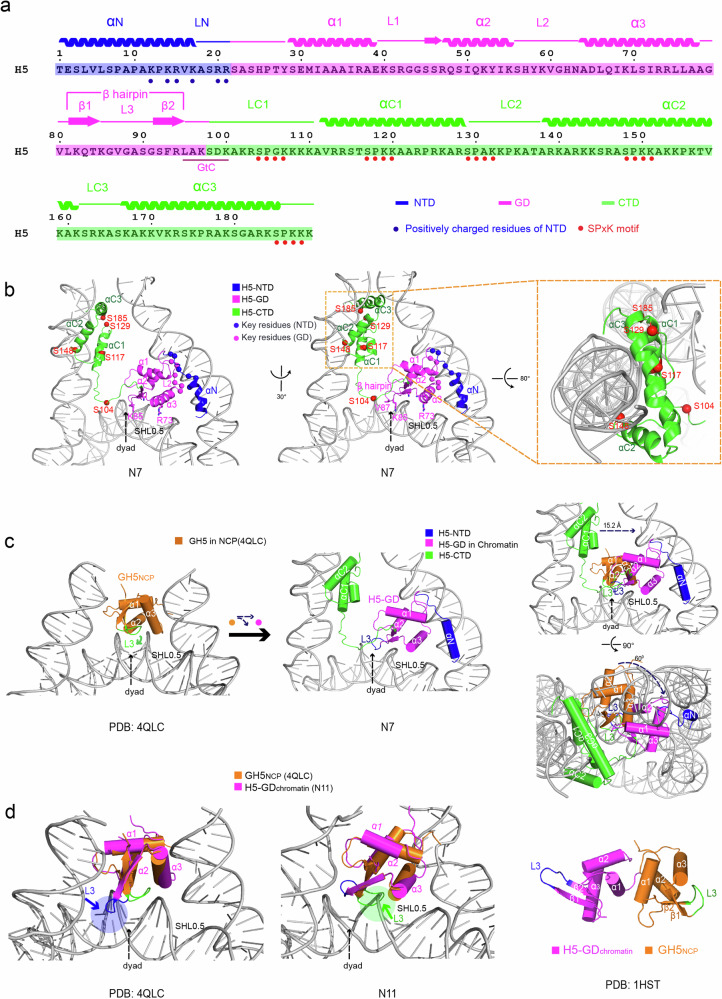
Structure of the full-length linker histone H5 and its binding to the nucleosomes in chromatin fiber. **a** The amino acid sequence of linker histone H5. NTD, GD, and CTD are colored blue, magenta, and green, respectively. The secondary structural regions as revealed by the structure of H5-chromatin, i.e., α-helices, β sheets, and loops, are marked. The locations of SPxK motifs and the positively charged residues in the NTD region are also indicated. **b** Left and middle: The overall structure of H5 in the N7 chromatosome of the chromatin fiber viewed from two different angles. The NTD, GD, and CTD of H5 are colored as in **a**. Residues of H5-GD close to nucleosomal dyad and linker DNA are labeled with sticks and magenta spheres, while the positively charged amino acids in H5-NTD and the serine residues in the SPxK motifs in H5-CTD are shown in blue and red spheres, respectively. Right: a zoomed-in view of the dashed box in the middle panel and viewed from a different angle showing the HMG box-like motif of the H5-CTD domain in the N7 chromatosome. See also Supplementary information Fig. S4. **c** Left and Middle: Comparison of the linker histone GD locations between the on-dyad binding mode in mono-nucleosome as shown in the crystal structure of GH5-NCP (PDB: 4QLC)^38^ (GH5_NCP_, orange, left) and the off-dyad binding mode in the chromatin fiber as shown in the chromatosome N7 of H5-chromatin (H5-GD_chromatin_, magenta, middle). The L3 loop of β-hairpin possesses a bent conformation (green) in the on-dyad binding GH5_NCP_ (orange) and an extended conformation (blue) in off-dyad binding H5-GD_chromatin_ (magenta), respectively. The nucleosome dyads are indicated. Right: From the on-dyad binding position in mono-nucleosome to the off-dyad binding position in chromatin, the H5 GD takes a conformational change with a shift of about 15.2 Å toward one side of the entry/exit linker DNA and a tilt of about 60 ° around the center of mass. **d** Left and Middle: Steric hindrances (highlighted with arrows) between the L3 loop of H5-GD and the nucleosomal DNA when the off-dyad H5-GD_chromatin_ (magenta with blue L3 loop) is aligned with its on-dyad counterpart, GH5_NCP_ (orange with green L3 loop) (left), and vice versa (middle). The nucleosome dyads are indicated. Right: The previously solved crystal structure of chicken GH5 alone (PDB:1HST)^60^ displays two conformers of H5 GD, e.g., a bent L3 loop (green) as in the on-dyad GH5_NCP_ (orange) and an extended L3 loop (blue) as in the off-dyad H5-GD_chromatin_ (magenta).

Strikingly, the structure of an intact chromatosome with full-length linker histone H5 can be clearly traced in our reconstructed cryo-EM density map of the H5-chromatin fiber. In two of the chromatosomes, i.e., N6 and N7, H5 can be traced in full length (from the 1st residue Thr1 to the last residue Lys189) (H5 in N7 shown in Fig. 1d). Based on the reconstructed cryo-EM density map, we built the atomic model of full-length linker histone H5 (Figs. 1d, 2b). Previously, it had been predicted that some secondary structures, e.g., α-helix, can be formed in the NTD and CTD tails of linker histone upon its binding to the DNA or nucleosome.^41,42^ Indeed, our cryo-EM structure reveals that upon binding to the entry/exit linker DNA, the linker histone H5 in each nucleosome of the H5-chromatin fiber adopts almost the same configuration (Supplementary information, Fig. S4a) with an obvious α-helix structure in its NTD which we term as αN, and three α-helices in its CTD which we term as αC1, αC2 and αC3, respectively (Fig. 2a, b). The three α-helices in H5-CTD are organized into an HMG box-like motif and tightly interact with one of the entry/exit linker DNA (Fig. 2b). Altogether, based on the resolved structure, the linker histone H5 can be divided into three individual structural parts, i.e., the H5-NTD which includes an α-helix (αN) and a loop (LN) connecting to the GD; the H5-GD containing three α-helices (α1, α2 and α3) flanked by two loops (L1 and L2), one β-hairpin composed of two β-sheets (β1 and β2) and a flanking loop (L3); and the H5-CTD which comprises a loop (LC1) connecting to the H5-GD, three α-helixes (αC1, αC2 and αC3) which form an HMG-like motif grabbing the entry/exit linker DNA, and two flanking loops (LC2 and LC3) (Fig. 2a, b).

### H5 binds to the nucleosome off-dyad through a three-contact mode in the chromatin fiber

As shown in Fig. 1c, H5 exhibits a 1:1 stoichiometric association with the nucleosome core in the H5-chromatin fiber, similar to what we previously observed in the H1.4-chromatin fiber.^21^ In each chromatosome, the histone H5 directly interacts with both the dyad and the entry/exit linker DNA through a three-contact mode with its NTD, GD, and CTD, respectively (Supplementary information, Fig. S4a). Specifically, the H5-GD contacts the nucleosomal DNA in an asymmetrical off-dyad manner, while the H5-NTD binds to one of the entry/exit linker DNA and the H5-CTD binds to the other linker DNA (Fig. 2b). In the off-dyad binding mode of H5-GD, the α3 helix sits on top of the major groove of nucleosomal dyad DNA at SHL+0.5 or SHL–0.5 location depending on the position of the nucleosome in H5-chromatin fiber, in which the residues Arg73 and Lys69 play critical roles in binding the dyad DNA; the β-hairpin lies on top of the dyad DNA, in which the residues Gln83, Lys85 and Val87 interact with the nucleosomal DNA on dyad; and the L2 loop locates next to one of the linker DNA (Fig. 2b; Supplementary information, Fig. S4a). Interestingly, the off-dyad location of H5-GD in the present structure of H5-chromatin fiber is in almost the same position and orientation of H1.4-GD as shown in our previous structure of H1.4-chromatin fiber^21^ (Supplementary information, Fig. S4b).

Previously, it had been reported that the NTD of the linker histone H1/H5 may help the GD in making proper contacts with the nucleosome and contribute slightly to its binding affinity to chromatin,^43–46^ while the role of NTD in the compaction of chromatin fiber remains elusive. In the structure of H5-chromatin fiber, 11 of the 12 H5 can be traced to the first residue Thr1, which enables us to build the structural model of the intact H5-NTD and to uncover the structural basis for its interactions with the nucleosomal/linker DNA. Notably, our structure shows that all the H5-NTDs adopt a similar α-helix structure (αN), which binds to one of the linker DNA at the entry/exit point of the nucleosome (Fig. 2b; Supplementary information, Fig. S4a). Among them, several positively charged residues (blue dots in Fig. 2b; Supplementary information, Fig. S4a) in H5-NTD are in close proximity with the linker DNA, providing an anchor point for H5 to bind the nucleosome in the three-contact mode. In addition, our structure shows that the N-terminal end of H5 is in close contact to one of the presumptive H3 N-tails in the nucleosome (Supplementary information, Fig. S3b–d), which suggests a possible interplay between the linker histone and the epigenetic modifications on histone H3 tails in the regulation of chromatin compaction and architecture as shown in recent studies.^47,48^

The CTD of the linker histone H1/H5, which weighs more than half of the total mass of the linker histone protein, plays a critical role in the folding of chromatin fiber.^30,44^ Due to the extensive positively charged residues, the CTD is mostly unstructured and intrinsically disordered in solution. Thus, structural information on the CTD is very limited, although it was predicted that some regional secondary structures, e.g., α-helix, can be formed upon binding to the nucleosome/DNA.^41,49^ Indeed, upon binding to the linker DNA, as shown in N7, the H5-CTD forms three α-helices in regions ~112–128, ~139–161, and ~165–185, i.e., αC1, αC2, and αC3, respectively (Fig. 2a, b; Supplementary information, Fig. S4a). These three helices are organized into an HMG box-like motif, which is commonly found in other DNA binding or chromatin architectural proteins to recognize the DNA structure,^50,51^ to tightly hold one of the entry/exit linker DNA (Fig. 2b). It suggests that the chromatin architectural proteins, including linker histone (H1/H5) and HMG group proteins which contain intrinsically disordered regions with a large number of positively charged residues, may adopt a common mechanism of nucleosome/DNA binding and chromatin compaction.

Posttranslational modifications in the linker histone, particularly, the phosphorylation at the conserved S/TPxK/R motifs of the H1/H5-CTD were found to play critical roles in chromatin folding during mitosis,^52–54^ however, the structural basis remains elusive. In the structure of H5-chromatin, all of the five S/TPxK/R motifs in H5-CTD (red dots in Fig. 2a) can be readily identified, while most of them are within the distance of electrostatic interaction with the linker DNA (Fig. 2b; Supplementary information, Fig. S4a). This suggests that phosphorylation in the S/TPxK/R motifs might neutralize the local positively charged net of the CTD and de-compact the chromatin fiber.^55–57^ Mutations in linker histone have been reported in different human diseases, e.g., frameshift mutations at the C-terminus of H1 isoform E in HIST1H1E syndrome (also known as Rahman syndrome)^58^ and missense mutations at the globular and CTDs of H1 isoforms (B, C, D, E) in B cell lymphomas.^59^ Our structure provides a structural basis by which these mutations in H1 could cause diseases through structural reorganization of the chromatin fiber, which leads to dysregulation of gene expression in B cells or neurons.^48^

### The switch from on-dyad to off-dyad binding of linker histone in the chromatin fiber

Similar to the location of H1.4-GD in our previous H1.4-chromatin structure,^21^ H5-GD was found to bind the nucleosome in an asymmetrical off-dyad mode in the H5-chromatin fiber (Fig. 2b; Supplementary information, Fig. S4a). Comparing the locations of H5-GD (GH5) in the mono-nucleosome bound complex (termed GH5_NCP_, PDB:4QLC)^38^ and in the H5-chromatin fiber (termed H5-GD_chromatin_), a lateral translocation of about 15.2 Å and a proximal tilt of about 60° on average is found from the on-dyad position to the off-dyad one (Fig. 2c). Although the location of the GD appears different when bound to the mono-nucleosome (on-dyad) or in the context of chromatin fiber (off-dyad), the overall structures of the H5-GD in these two different binding modes are almost identical except the conformations of L3 loop within the β-hairpin (Fig. 2d, left and middle panels). The L3 loop in the β-hairpin of the on-dyad binding GH5_NCP_ is bent and folded back toward itself, whereas this loop is extended and binds the dyad-DNA in the off-dyad binding H5-GD_chromatin_ (Fig. 2c, d). Strikingly, the previously solved crystal structure of H5-GD alone (PDB: 1HST)^60^ showed exactly the same two conformations of L3 loop (Fig. 2d, right panel), i.e., the folded conformation (named as conformation A) as found in the GH5_NCP_, and the extended conformation (conformation B) as found in the H5-GD_chromatin_, respectively.

Notably, a steric hindrance between the L3 loop and the nucleosomal DNA could be clearly observed if the on-dyad conformation A in the GH5_NCP_ were substituted with the off-dyad conformation B in the H5-GD_chromatin_ fiber and vice versa (Fig. 2d, left and middle panels). Considering the high conservation in the GD of linker histones across different species or variants (dashed box in Supplementary information, Fig. S1a), these results suggest that the two conformations of the L3 loop appear to play a key role in arranging the nucleosomal arrays from an open “beads-on-a-string” conformation to a compacted chromatin fiber. This process is akin to acting as a brake paddle in the switch from the on-dyad to the off-dyad binding mode of the linker histone during the folding of chromatin fiber (Fig. 2c, d).

Similar to our previous study of H1.4-chromatin fibers,^21^ the reconstituted H5-chromatin fibers were cross-linked by 0.2% glutaraldehyde (GA) to prevent the potential disassociation of the chromatosome and deformation of the reconstituted chromatin fiber during the cryo-EM sample processing. To verify whether the GA fixation would alter the binding mode of H5 to the nucleosome as previously speculated,^39,61^ we prepared the reconstituted mono-nucleosomes with 167 bp of 601 DNA in complex with either the GH5-NCP or full-length H5 (H5-NCP), cross-linked them with 0.2% GA as we did in the H5-chromatin fiber, and performed the cryo-EM single-particle reconstructions. The 3D cryo-EM structures of the chemically fixed H5-NCP and GH5-NCP were acquired at the resolutions of 3.3 Å and 3.6 Å, respectively (Supplementary information, Fig. S5). Notably, both cryo-EM structures show an apparent on-dyad binding of the H5 GD to the nucleosome, which is consistent with previous observations in the crystal structure of the GH5-NCP^38^ and the cryo-EM structure of the NCP-H1.0^39^ without GA fixation (Supplementary information, Fig. S6). These results suggest that the GA fixation did not alter the binding mode of H5 GD to the nucleosome. Instead, it implies that the switch of binding mode of linker histone H1/H5 to the nucleosome, i.e., from on-dyad binding in the H5-NCP to off-dyad binding in the H5-chromatin fiber (Fig. 2c, d), is likely due to the constraint of linker DNA, such as the asymmetric geometry and orientation of linker DNA during the folding from the open nucleosome arrays to the higher-order chromatin fiber as discussed previously.^39,62,63^

Recently, Cramer lab reported the cryo-EM structures of tetranucleosomes with different linker DNA lengths in the presence of full-length linker histone H1.4. They found that linker DNA length impacted the stoichiometry of H1:nucleosome. Interestingly, similar to that in mono-nucleosome, they observed an on-dyad association of H1.4 in the tetranucleosome, even with the 177-bp repeat length.^40^ These results suggest the association mode of linker histone is relevant to the context/constraint of the chromatin fiber, e.g., the inter-tetranucleosomal interactions and the H1–H1 self-association between tetranucleosomes, may play a role in the association mode of linker histone, or vice versa.

### The nucleosome–nucleosome interactions in the chromatin fiber

**Fig. 3:**
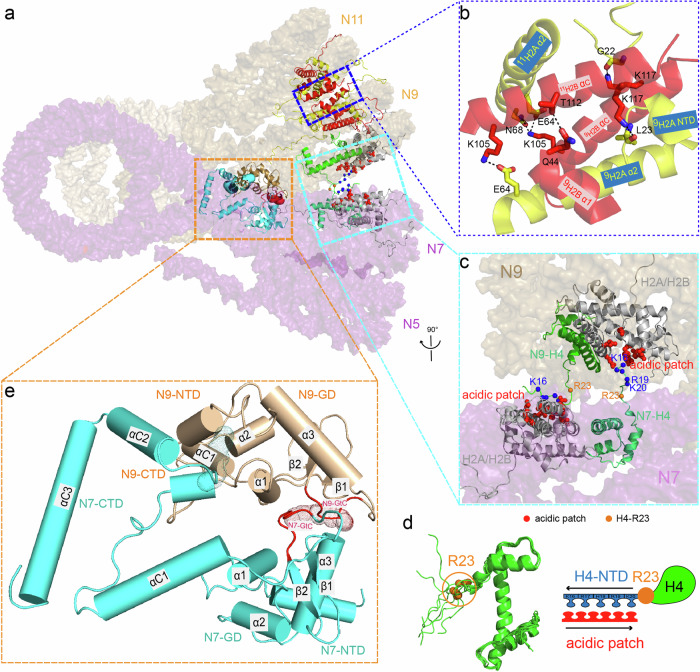
Intra- and inter-tetranucleosomal interactions in the H5-chromatin fiber. **a** Two tetranucleosomal units of the H5-chromatin are displayed to exhibit the interactions within and between tetranucleosomal units. The regions enclosed by the blue, cyan, and orange dashed lines are illustrated in detail in **b**, **c** and **e**, respectively. **b** The structural details of nucleosome–nucleosome interactions within tetranucleosomal unit engaged by adjacent H2A–H2B and H2B–H2B pairs, in which H2A (yellow) and H2B (red) are shown in ribbon representation. The key residues involved in the interactions are labeled and shown in sticks. See also Supplementary information, Fig. S8a and Table S1. **c** The structural details of the nucleosome–nucleosome interactions between tetranucleosomal units engaged by H4 N-tails (green) and the adjacent H2A-H2B acidic patches (red dots). The key residues involved in the interactions on H4 N-tails are numbered and highlighted by blue spheres. **d** Left: the structural alignments of the H4 N-tails participating in the interactions between tetranucleosomal units. The hinge residue, R23, is highlighted. Right: a “zipper” model for the interactions between H4 N-tails and the adjacent acidic patches. **e** The structural details of the H5–H5 interaction between tetranucleosomal units. The meshed dots show the interaction regions. See also Supplementary information, Fig. S8b.

Previously, we found that the interactions between adjacent nucleosomes, both within the tetranucleosomal units (type I) and between the tetranucleosomal units (type II), and the H1–H1 interactions between adjacent tetranucleosomal units play important roles in the formation and stabilization of the chromatin fiber.^21^ Although with a little more compacted configuration as shown by the relatively larger twisted angles (Supplementary information, Fig. S7), the 12-mer H5-chromatin fiber also comprises three twisted tetranucleosomal units and presents almost the same overall architecture as the H1.4-chromatin. The above-mentioned nucleosome–nucleosome interactions were also found to be critical in the H5-chromatin fiber (Fig. 3). Notably, based on the structure of the H5-chromatin fiber with the significantly improved resolution (3.6 Å), much more structural details for the nucleosome–nucleosome interactions within or between tetranucleosomal units can be uncovered.

### The intra-tetranucleosomal interactions

The two nucleosomes in each stack directly contact each other through multiple H2A–H2B and H2B–H2B interactions (Fig. 3a, b; Supplementary information, Fig. S8a). Overall, three distinct classes of H2A–H2B and H2B–H2B interaction patterns, like three bridges linking two adjacent nucleosomes, were observed in the six pairs of intra-tetranucleosomal nucleosome–nucleosome interactions, i.e., H2B-αC with the adjacent H2A-α2, H2B-αC with adjacent H2B-α1, and H2B-αC with the adjacent H2A-NTD, respectively (Supplementary information, Fig. S8a). Although the nucleosome–nucleosome interaction modes and interfaces are largely similar among the three tetranucleosomal units, the key residues involved in these interfaces are somewhat variable among the six different nucleosome–nucleosome pairs (Supplementary information, Table S1). Among them, the molecular interactions between K105H106 of H2B-αC and E64/N68 of adjacent H2A-α2 as Bridge I, S109 and T112 of H2B-αC and Q44 of adjacent H2B-α1 as Bridge II, K113/K117 of H2B-αC and G22/L23 of the adjacent H2A-NTD as Bridge III are most frequently observed, mainly through electrostatic and hydrogen bond interactions (Supplementary information, Fig. S8a and Table S1). Notably, various mutations in these key residues involved in nucleosomal interactions within tetranuclesomal units were found to be enriched in the oncogenic histone mutations (named oncohistone) correlated to multiple human cancers.^64^

### The inter-tetranucleosomal interactions

Our previous cryo-EM structures of the H1.4-chromatin fiber revealed that the tetranucleosomal units are connected and twisted by three interactions: a pair of interactions between H4 N-tails and its adjacent nucleosome’s acidic patches and the H1.4–H1.4 interaction.^21^ Similarly, in the present structure of the H5-chromatin fiber, a pair of interactions between the N-terminal tails of H4 and the acidic patches of the adjacent H2A–H2B dimer are also identified in the nucleosome–nucleosome interface between tetranucleosomal units (Fig. 3a, c). Notably, with the improved reconstruction resolution (3.6 Å), we can trace and model the majority of the H4 N-terminal tails, showing that the H4 tails extend from the nucleosome core to contact with the acidic patches of the adjacent H2A–H2B dimers (Fig. 3c).

Remarkably, by aligning all of the resolved H4 N-terminal tails participating in the inter-tetranucleosomal interactions in the H5-chromatin fiber, we observe a noteworthy flexibility among the location and orientation of the H4 N-terminal tails, while the residue R23 remains at a relatively rigid position (Fig. 3d, left panel). This result suggests that R23 acts as a hinge residue which allows the positively charged H4 tails to adopt flexible configurations for interacting with the acidic patches on the adjacent H2A–H2B dimers. Thus, we hypothesize that the positively charged amino acid residues in the H4 N-tails, with R23 acting as a hinge point, interact with the negatively charged residues on the adjacent acidic patches dynamically in a zipper mode, in which the interactions mainly depend on the amino acid composition, other than a rigid/static residue-to-residue match (Fig. 3d, right panel).

### The H5–H5 interactions between tetranucleosomal units

Linker histone self-associations have been reported to play an important role in the formation and maintenance of the chromatin fiber.^65–67^ Previously, our cryo-EM structures of the H1.4-chromatin revealed an unknown arrangement of H1–H1 self-association attributed to the interaction between the GDs of adjacent linker histones (GH1–GH1 interaction), which imparts an additional twist between tetranucleosomal units.^21^ However, the molecular details for this self-association of the linker histone are still ambiguous due to the relatively low resolution. In our current cryo-EM structure of the H5-chromatin fiber, four pairs of H5–H5 interactions, i.e., N3–N5, N4–N6, N7–N9, and N8–N10, can be identified with an interface area of about 995 Å^2^, 1551 Å^2^, 972 Å^2^ and 316 Å^2^, respectively, measured using Qt-PISA,^68^ indicating that H5–H5 self-associations are quite abundant between distinct tetranucleosomal units (The H5–H5 interface between N8 and N10 is measured smaller than the others as a large part of H5-CTD structure of N8 is not well resolved). These four pairs of H5–H5 self-associations display remarkably similar molecular interactions (Fig. 3e; Supplementary information, Fig. S8b), in which a loop region with 6 amino acid residues (LAKSDK) located between the H5-GD and the H5-CTD (termed as GtC loop as indicated in Fig. 2a) is frequently observed to interact with the GD of the adjacent H5 (Fig. 3e; Supplementary information, Fig. S8b). Moreover, the GD-CTD and NTD-CTD interactions between the adjacent nucleosome pairs are also observed in the H5–H5 self-associations between tetranucleosomal units (Fig. 3e; Supplementary information, Fig. S8b).

### The tetranucleosomal folding related residues are critical for cell growth and gene transcription in budding yeast

**Fig. 4:**
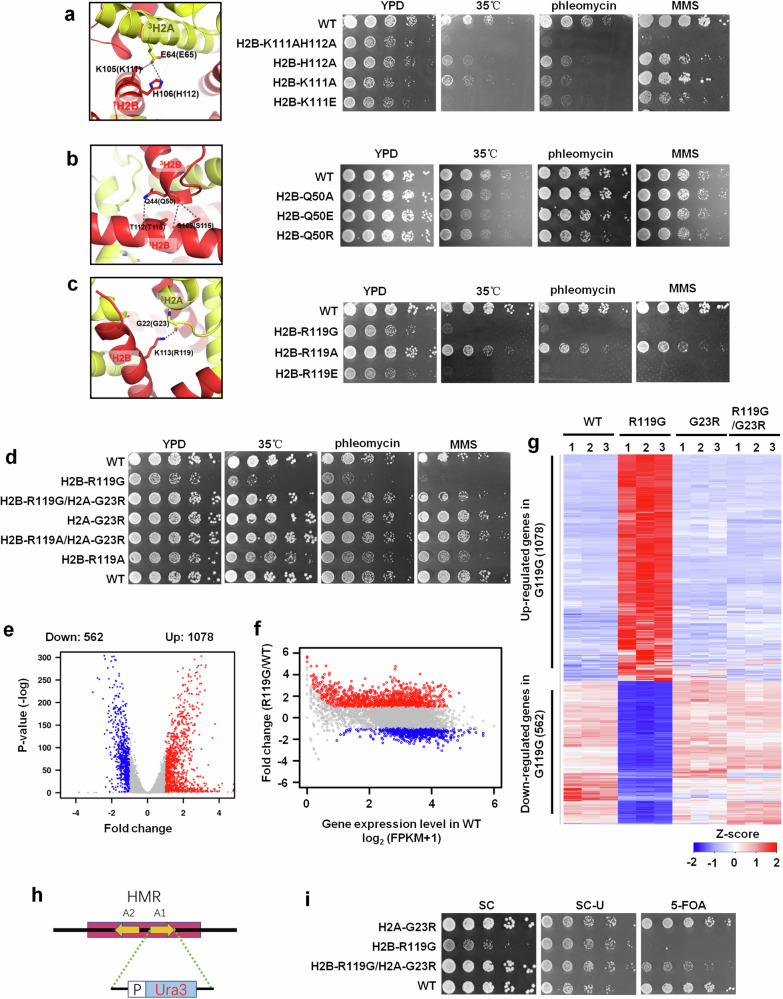
Effects of histone mutations destabilizing the tetranucleosome on growth and gene transcription in budding yeast. **a**–**c** Structure details of the most frequent interactions within the tetranucleosome and yeast growth defect resulting from different mutations of the residues involved in these interactions. Spot dilution growth assays with 5-fold serial dilutions. Mutants carrying these mutations exhibit increased sensitivity to temperature stress and DNA damage stress, as compared to WT strains. YPD, yeast extract, peptone, dextrose full medium; phleomycin (1 μg/mL); MMS (0.04%). **d** Spot dilution growth assays test the viability of yeast with mutations of H2B-R119 with/without H2A-G23R. **e** Volcano plots depict the gene expression changes of H2B-R119G compared to WT. The red dots represent significantly up-regulated genes with a log_2_ (fold change) ≥ 1 and *P*-value ≤ 0.05. The blue dots represent significantly down-regulated genes with a log_2_ (fold change) ≤  –1 and *P*-value ≤ 0.05. **f** MA plot showing the expression level for significantly differentially expressed genes. The red and blue dots represent the up- and down-regulated genes same as **e**. **g** Heatmap indicates the changes in gene transcription. Each strain was replicated three times. The heatmap shows the differential gene expression between WT and mutant strains, indicating that the histone mutations affect the transcription of many genes. **h** Position and transcriptional orientation of the inserted *URA3* gene on *HMR* loci. **i** Assays for silencing of the *URA3* gene inserted on *HMR* loci of the WT and mutant strains by monitoring the cell growth on the synthetic complete plate without uracil or with 0.1% 5-FOA.

As a classical model organism, budding yeast *Saccharomyces cerevisiae* has a unique advantage in the genetic analysis of histones and their roles in the regulation of chromatin organization and gene transcription, as it has only two copies of each histone genes and their protein sequences are highly similar to mammalian ones. To substantiate the physiological functions of the tetranucleosomal folding unit, we generated various mutations in those key residues on histone H2A-H2B that are involved in stabilizing the tetranuclesomal units (Fig. 3b; Supplementary information, Fig. S8a and Table S1) through the plasmid-shuffling scheme in *S. cerevisiae*, as previously reported.^69^ First, we widely screened these histone H2A-H2B mutants for phenotypic assays by spot tests on YPD plates or plates with different media (drugs used including CPT, phleomycin, MMS (methyl methanesulfonate), and HU, or treating at high temperatures at 35 °C and 37 °C). We observed that most mutations cause various degrees of growth defects or increased cell sensitivities to DNA damages, especially the mutations related to the three interaction bridges: Bridge I for interactions between H2B-K111H112 and adjacent H2A-E65N69 (i.e., H2B-K105H106 and H2A-E64/N68 in *Xenopus laevis*), Bridge II for interactions between H2B-S115T118 and adjacent H2B-Q50 (i.e., H2B-S109T112 and H2B-Q44 in *X. laevis*) and Bridge III for interactions between H2B-R119K123 and adjacent H2A-G23 (i.e., H2B-K113K117 and H2A-G22 in *X. laevis*) (Fig. 4a–c; Supplementary information, Tables S2, S3). In particular, mutating two key residues K111H112 on Bridge I (H2B-K111H112) to acidic residues (H2B-K111EH112E) proved lethal (Supplementary information, Fig. S9b). According to the structure, these mutations could generate a strong repulsive force and intensively destabilize the tetranucleosome structure. Similarly, the mutation of H2B-K111AH112A which can impair the interactions within the tetranucleosome also reduced the viability of yeast (Fig. 4a). Of note, even single-residue mutations (H2B-H112A, H2B-K111A, H2B-K111E, and H2B-H112E) were severely detrimental (Fig. 4a; Supplementary information, Table S3). Similarly, mutations of the key residue R119 of H2B on the Bridge III (H2B-R119G and H2B-R119E) also caused severe growth defects, although H2B-R119A displayed less growth defects compared with H2B-R119G or H2B-R119E (Fig. 4c; Supplementary information, Table S3).

Meanwhile, mutations of the key residues on Bridge II which is located in the middle of the intra-tetranucleosomal interface, had a much weaker phenotypic effect on cell growth and sensitivity to DNA damage (Fig. 4b). Interestingly, compared with impairing the single-interaction bridge, breaking multiple-interaction bridges was more harmful or even lethal (Supplementary information, Fig. S9c and Table S3). Taken together, all these results suggest that the tetranucleosomal folding-related residues play an important role in cell growth and response to DNA damage.

To further validate whether the cell growth defect phenotype is caused by impairment of the tetranucleosome, rather than the effect on the nucleosome level, we performed the rescue experiments by exchanging the two key resides on Bridge III (H2A-G23R and H2B-R119G double mutation). Intriguingly, we observed that the growth defect caused by impairment of Bridge III (H2B-R119G or H2B-R119A mutation) was almost fully rescued to wild-type (WT) level by restoring the bridge III through introducing a compensatory mutation on H2A (H2A-G23R) (Fig. 4d; Supplementary information, Table S3). This result convincingly supports that the tetranucleosomal folding-related residues play important regulatory roles in genome folding and genome functions in yeast.

To explore the role of tetranucleosomal folding unit in transcription regulation, we carried out RNA-sequencing (RNA-seq) on both histone mutant and WT strains (Supplementary information, Fig. S9d). Compared with WT strain, we identified 562 down-regulated and 1078 up-regulated genes in the H2B-R119G mutant strain (Fig. 4e). In line with the influence of H2B-R119G mutation on the compaction of the tetranucleosome, the up-regulated genes mainly belong to the lowly expressed genes (Fig. 4f). Of note, the up-regulated genes exhibit lower levels of active histone marks, including H3K4me3 and H3K9ac, while they show higher levels of histone mark H3K4me1 which has been reported to be related to gene repression and expression activation under stress in yeast^70,71^ (Supplementary information, Fig. S9e). Interestingly, consistent with the results of phenotypic assays, the defective transcriptions in the H2B-R119G mutant strain can be rescued to the same level as the WT strain by the double mutation H2B-R119G/H2A-G23R (Fig. 4g; Supplementary information, Fig. S9d). Together, these results show that impairment of tetranuclesomal folding units may result in the upregulation of the repressive genes by disrupting the compacted chromatin. In addition, similar transcriptional changes are found in both the H2B-K111AH112A and H2B-R119E mutant strains as observed in the H2B-R119G mutant strain (Supplementary information, Fig. S9f). Intriguingly, substantial overlaps were observed in transcriptional changes among these three distinct mutants that destabilize the tetranucleosome (Supplementary information, Fig. S9g). Further, GO analysis suggested that the up-regulated genes, which are primarily involved in protein folding or refolding processes, may affect the cell growth and response to DNA damage in yeast (Supplementary information, Fig. S9h).

To further elucidate the role of the tetranucleosomal unit in chromatin compaction and gene transcription, we constructed a reporter system by inserting a *URA3* gene, whose transcriptional silencing can be tested by the growth of yeast cells on the plate with uracil treatment (Fig. 4h). Similar to the cell growth phenotype and RNA-seq assays, our results showed that the histone H2A-H2B mutations destabilizing the tetranucleosome caused an increased transcription level of *URA3* compared with the WT (Supplementary information, Fig. S9i). Moreover, the activated transcription of *URA3* in the H2B-R119G mutant strain was restored to a repressive state by the compensatory H2A-G23R mutation, which can repair the intra-tetranucleosomal interaction on Bridge III (Fig. 4i). All together, these findings suggest that the tetranucleosome, as an intermediate structural unit of the 30-nm chromatin fiber, plays a critical role in transcriptional regulation.

### The effect of histone H2A-H2B mutations on the tetranucleosomal folding unit in vivo and in vitro

**Fig. 5:**
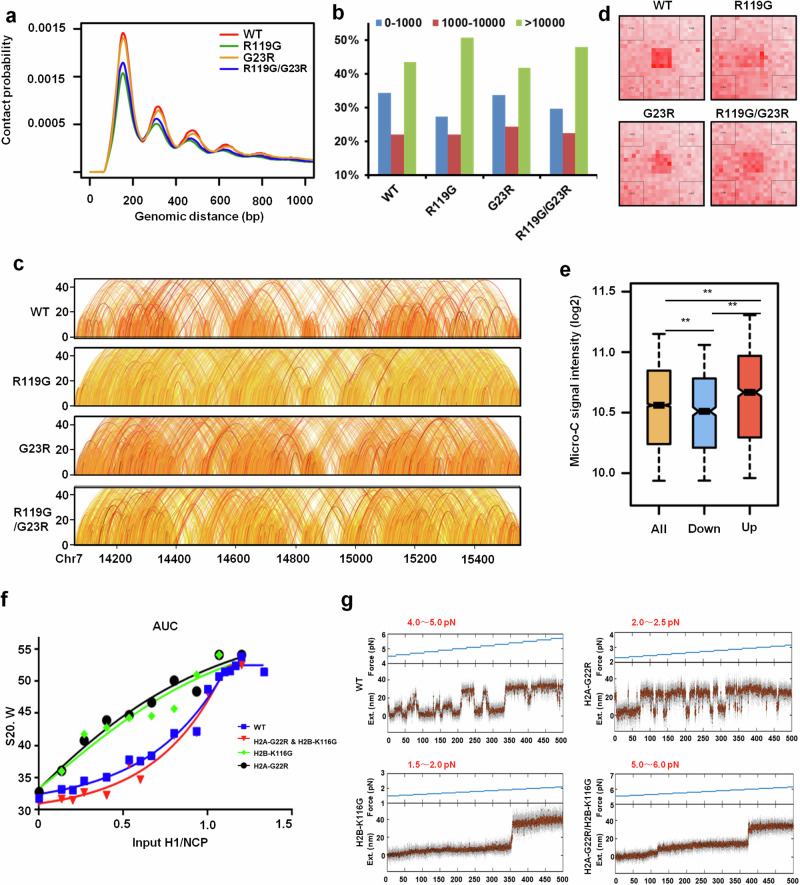
Destabilized tetranucleosome impacts the whole structure of chromatin fiber. **a** Decay of product abundance with distance is shown separately for micro-C products with read pairs facing toward OUT-IN and IN-OUT within 1 kb. These plots show the differences in nucleosome interaction frequencies between WT and mutant strains. **b** Effect of the mutants on interaction frequency at different scales. The color scheme corresponds to the plots in **a**. These data further illustrate the impact of the histone mutations on chromatin structure. **c** Data from different strains for a 15 kb locus, with arcs showing interactions between nucleosomes. The interactions represented in white-yellow-red arcs show the interaction intensity for a given pair of loci. **d** Aggregate plots for nucleosome interaction changes in different mutants compared to WT. **e** Boxplot showing the micro-C signal intensity for all, down-regulated and up-regulated genes in R119G mutant. **f** The plot of S20 mid-points of 12× 177 bp nucleosome array in the presence of WT, H2A-G22R, H2B-K116G and H2A-G22R/H2B-K116G with different amounts of histone H1e. **g** Unfolding Dynamics of Tetranucleosomal Units Analysis in the presence of histones same as **f** using 4× 177 bp nucleosome array.

To further understand the direct impact of the aforementioned histone mutations on the tetranucleosome stability and chromatin compaction in vivo, we carried out micro-C assays to examine chromosome folding in the relevant mutant strains.^24^ Contact probability analysis revealed that, in the H2B-R119G mutant strain, short-range interactions (N/N + 1 or N/N + 2) are significantly decreased, while long-distance interactions are increased compared with those in the WT strain (Fig. 5a–c). Similar patterns were observed in mutants H2B-R119A and H2B-K111AH112A (Supplementary information, Fig. S10a, b). Notably, Aggregate Peak Analysis (APA) plots showed that, compared to the WT strain, the R119G mutant decreased the conserved loop strength (Fig. 5d), despite the long-distance interaction increased. Besides, compared to the micro-C signals on the upregulated and downregulated genes in the R119G mutant, our results showed that the upregulated transcription mainly occurred in the repressive genes with more compacted chromatin structures (Fig. 5e). These results provide compelling evidence of dysregulation in the 3D structure across the entire genome, which aligns with our observations from the transcriptome analysis. Interestingly, the double mutant H2B-R119G/H2A-G23R can partially rescue these chromatin compaction defects (Fig. 5a–d). These results suggest that the tetranucleosome is an essential folding unit of chromatin compaction and genome folding in yeast, which plays an important role in regulating gene transcription and/or other DNA-related biological processes.

To further explore the direct impact of the aforementioned histone mutations on the tetranucleosome stability and chromatin compaction, we also conducted a battery of in vitro biochemical and biophysical experiments, using AUC and single-molecule magnetic tweezer analyses. To confirm that the interactions observed in the H5-bound chromatin fiber are consistently present in the H1-bound chromatin fiber, we substituted H5 with H1 in the AUC assay. Our AUC analyses showed that compared with the WT nucleosomal arrays, either the H2B-K116G (i.e., H2B-R119G in yeast) or the H2A-G22R (i.e., H2A-G23R in yeast) arrays exhibit much different levels of compaction as indicated by S20 distribution, particularly at the lower ratios of liner histone to nucleosome (Fig. 5f), while H2B-K116G/H2A-G22R arrays show almost the same compaction manner with the WT arrays (Fig. 5f; Supplementary information, Fig. S10c). Consistently, in the negative staining EM, the particles with mutants display a less-twisted configuration compared to WT particles, resembling more of a ladder pattern. These results suggest that both the H2A-G22R and the H2B-K116G mutation impair the in vitro chromatin compaction by destabilizing the intra-tetranucleosomal interactions (Bridge III) and the broken interaction can be restored by the residue exchange. Previously, we employed single-molecule magnetic tweezers to monitor the folding and unfolding dynamics of the tetranucleosome and its regulation by the histone chaperone FACT in vitro.^22^ We performed the single-molecule magnetic tweezer measurement and showed that either the H2A-G22R or the H2B-K116G mutation lowers the force for unfolding the tetranucleosome (Fig. 5g), indicating these two single mutations directly impair the stability of the tetranucleosome. By contrast, the double mutation H2A-G22R/H2B-K116G almost fully restore the stability of the tetranucleosome to the WT level. All these results are largely consistent with the findings in our cryo-EM structure and the effects observed in vivo. In addition, mutation of either H2B-K108E (i.e., H2B-K111E in yeast) or H2B-Q47R (i.e., H2B-Q50R in yeast) can also lower the stability of the tetranucleosome (Supplementary information, Fig. S10d). In summary, these results show that mutations of the key residues involved in the intra-tetranucleosomal interactions may impair the tetranucleosome folding and chromatin compaction, leading to dysregulation of transcription and ultimately resulting in growth defects in yeast.

### Structural asymmetries of the core histone tails and the polarity of the H5-chromatin fiber

**Fig. 6:**
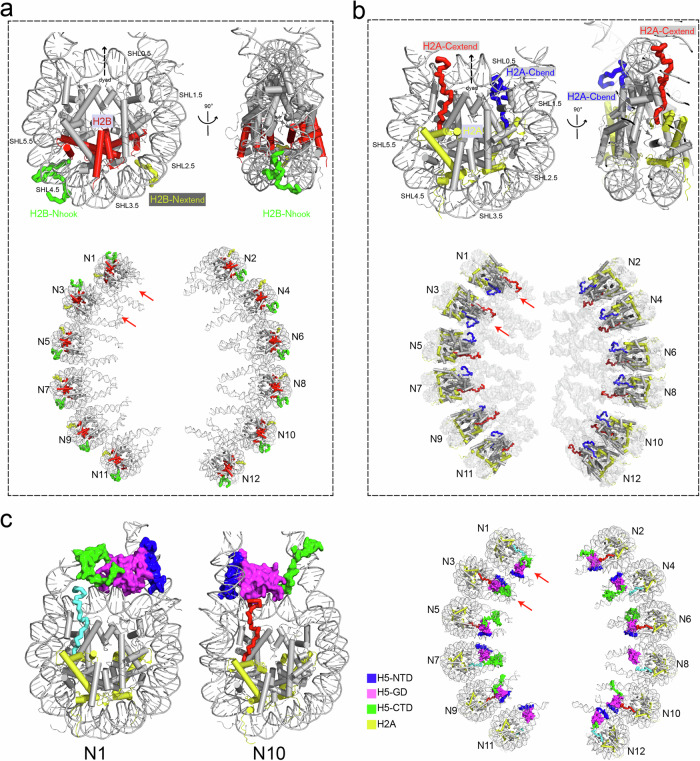
Structural asymmetries of H2B N-tails and H2A C-tails confer a polarity to the chromatin fiber. **a** Top: two different structural conformations of the H2B N-tails, i.e., H2B-N_extend_ (yellow) and H2B-N_hook_ (green), in the chromatosome are viewed from two angles. Down: the unidirectionally tandem arrangement of the H2B N-tails, except N1 and N3 as indicated by arrows, in the H5-chromatin fiber. All elements in the nucleosome are shown in grey except the two copies of H2B (red). **b** Top: two different structural conformations of H2A C-tails, i.e., H2A-C_extend_ (red) and H2A-C_bend_ (blue), in the chromatosome are viewed from two angles. Down: the unidirectionally tandem arrangement of the H2A C-tails, except N1 and N3 as indicated by arrows, in the H5-chromatin fiber. All elements in the nucleosome are shown in grey except the two copies of H2A (yellow). **c** Interplays between H2A-CTD and H5 in the chromatin fiber. Left: the relative positions of H2A C-tails and H5. The extended H2A C-tail in each nucleosome of the chromatin fiber points either to H5-CTD (N1) or to H5-GD (N10), depending on the positions of nucleosomes in the chromatin fiber. Right: the unidirectionally tandem arrangement of the asymmetric interactions between the H2A C-tails and the H5, except N1 and N3 as indicated by arrows, in the H5-chromatin fiber. The extended H2A C-terminal tails pointing toward H5-CTD are shown in cyan while those pointing toward H5-GD are shown in red.

As mentioned above, almost all of the core histones with majority of the histone tails can be traced and modeled in our cryo-EM structure of H5-chromatin fiber (Fig. 1b, d). Strikingly, the two copies of core histone tails, e.g., H2B-N tails, and H2A-C tails, exhibit apparently asymmetric conformations with a noteworthily patterned orientation in each of the nucleosome along the chromatin fiber (Fig. 6).

In each nucleosome of the H5-chromatin fiber, the two copies of H2B display a clearly asymmetric structural conformation in the N-terminal tail, i.e., one N-terminal tail of the H2B extends from the gap between the nucleosome DNA gyres and folds back to grip one of outwrap DNA gyre (termed as H2B-N_hook_), while the other N-tail of H2B swings flexibly with a relatively extended density (termed as H2B-N_extend_) which lacks the density of ~20 residues compared with the H2B-N_hook_ (Fig. 6a, top panels). The hooked H2B N-tails in the dodecanucleosome, except for N1 and N3, are arranged in a unidirectionally orientated pattern along the two-start chromatin fiber, i.e., the hooked H2B N-tails in individual nucleosomes display the same orientation along the dodecanucleosome of the two-start chromatin fiber (Fig. 6a, bottom panel). Remarkably, this unidirectionally patterned orientation of asymmetric H2B N-tails confers a polarity to the chromatin fiber.

Similarly, the two C-terminal tails of H2A in each nucleosome also display apparent structural asymmetries with different conformations, i.e., one H2A C-tail exhibits an extended conformation (termed H2A-C_extend_) while the other one adopts a bent conformation (termed H2A-C_bend_) (Fig. 6b, top panels). Consistent with those of H2B N-tails, the extended H2A C-tails, except for N1 and N3, also form a unidirectional orientation pattern in the H5-chromatin fiber (Fig. 6b, bottom panel). The extended H2A C-tail, i.e., H2A-C_extend_ tail, stretches out from the nucleosome core and extends toward the linker histone, where it approaches the off-dyad bound H5 at either the GD (H5-GD) or CTD (H5-CTD), depending on the location of that nucleosome in the H5-chromatin fiber (Fig. 6c, left two panels). It is worth noting that these featured interplay patterns between H2A C-tails and the linker histones are also arranged in a unidirectional orientation pattern with N1 and N3 as two exceptions (Fig. 6c, right panel), which may play critical roles in the binding of linker histone and compaction of the chromatin fiber.

The asymmetric conformation and unidirectionally patterned orientation are also observed for most of the N-terminal tails of H4, i.e., one H4-N tail exhibits up-ward conformation (termed as H4-N_up_) while the other one shows down-ward conformation (termed as H4-N_down_) in the H5-chromatin fiber (Supplementary information, Fig. S11). It should be noted that similar asymmetric conformation of two copies of H2B N-tails, H2A C-tails, and H4 N-tails had been previously observed in the crystal structure of nucleosome core particle with the most histone tails resolved (PDB: 1KX5),^72^ which likely resulted from crystal packing. These results suggest that the structural asymmetries of histone tails within the individual nucleosome likely resulted from the nucleosomal stacking in the chromatin fiber, while the asymmetric conformation and unidirectionally patterned orientation of histone tails in the nucleosome may confer the polarity to the chromatin fiber. Taken together, our structure suggests that structural asymmetries of the core histone tails and their interplay with the off-dyad bound linker histone, may stabilize the nucleosome in an asymmetrical manner and confer a polarity to the chromatin fiber. We suggest that the linker histones H1/H5 likely bind to nucleosomes in an on-dyad manner in previous structural reports, due to that the chromatin was reconstituted with less than two tetranucleosomes, i.e., eight nucleosomes, and thus there was a lack of interactions between the tetranucleosomes.^40^

### Polarities of the nucleosome and the folding of the chromatin fiber

**Fig. 7:**
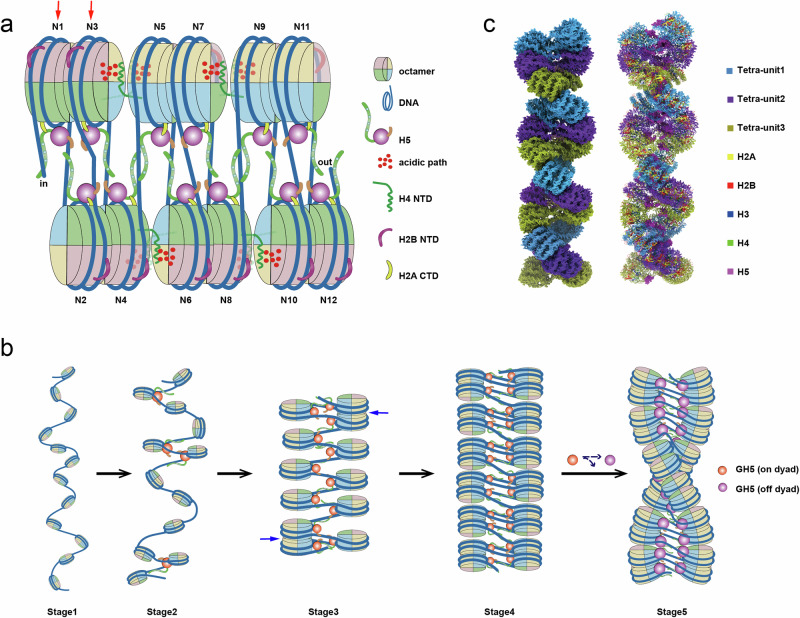
Structural insights and an assembly model of the H5-chromatin fiber. **a** An illustrated cartoon diagram displaying the structural insights into H5-bound dodecanucleosomal fiber. The structural asymmetries of H2B-NTD (purple), H2A-CTD (yellow), and the interplay between the extended H2A C-tails with either H5-GD (magenta) or H5-CTD (green) are highlighted by different colors, conferring a polarity to the chromatin fiber. N1 and N3 show an opposite polarity as indicated by red arrows. H5 (NTD: orange, GD: magenta, CTD: green) binds to the nucleosome off-dyad in a three-contacts mode, while the H5 of two adjacent stacked nucleosomes are arranged with a “head-to-head” polarity within the tetranucleosome unit. The tetranucleosomal units are connected by a pair of interactions between H4 N-tails (green) and their adjacent acidic patches (red dots), and twisted through the H5–H5 interactions between tetranucleosome units (not shown for viewing purposes, see **b**). **b** A model illustrating an assembly pathway from the open nucleosomal arrays into the compacted chromatin fiber with a two-start helical structure twisted by tetranucleosomal units. The switch from on-dyad binding (orange) to off-dyad binding (magenta) of H1/H5 (as shown in Fig. 2c) plays a critical role in the assembly of the chromatin fiber. **c** A density map of the chromatin fiber with 48 nucleosomes and 177-bp nucleosome repeat lengths (left) and its corresponding atomic model (right), built by directly stacking the cryo-EM structure of the dodecanucleosomal H5-chromatin fiber on top of each other to form a continuous fiber.

The H5-chromatin fiber structure showed that the GD of H5 binds the nucleosome in off-dyad mode and the N- and C-terminal tails of H5 are arranged asymmetrically with respect to the dyad of the nucleosome (Fig. 2b–d; Supplementary information, Fig. S4a). According to the asymmetric distributions of the core histone tails, e.g., H2B-N and H2A-C, and the locations of the H5-NTD and H5-CTD, the chromatosomes/nucleosomes in the H5-chromatin fiber could be conferred to different polarities, e.g., “head” or “tail” polarity, respectively (Figs. 1d, 6 and 7a). Within a tetranucleosomal unit, the two adjacent stacked nucleosomes are arranged with the opposite (head-to-head) polarity of the H5 location (Fig. 7a). This specific arrangement of the H5 polarity within the tetranucleosomal unit yields a different spatial arrangement and distribution of CTD-bound linker DNA, which consequently results in the H5–H5 interaction between the tetranucleosomal units. The dimerized linker histones within the fiber thereby could stabilize coherent stacking and twisting of the tetranucleosome units to form a two-start left-handed chromatin fiber (Fig. 7b).

Ideally, the nucleosomal arrays are oriented in tandem, and then they could be folded into a perfect two-start chromatin fiber with a unidirectional asymmetry and polarity. Unexpectedly, our structure shows that compared with the other 10 nucleosomes, the N1 and N3 nucleosomes in the tetra-unit I display an opposite polarity, conferred by the structural symmetries of H2A C-tails and H2B N-tails, indicating that these two stacked nucleosomes (N1 and N3) take a 180° flip concurrently (Figs. 6 and 7a, red arrows), although the binding mode of N1–N3 stack pair remains almost the same as in other nucleosome pairs within the tetranucleosome unit (Supplementary information, Table S1). Consequently, the linker DNA between the N2 and the N3 enforces a kinked conformation with a large deformation of B-form DNA, showing a conflict arrangement of major and minor grooves in the center (Supplementary information, Fig. S12).

## Discussion

We propose a model and principle for the folding of the chromatin fiber, as shown in Fig. 7. First, in the absence of linker histone H1/H5, the nucleosomal array adopts an open configuration because of repulsion between the negative charge of linker DNA, i.e., “beads-on-a-string” state (Fig. 7b, Stage 1). When the linker histone H1/H5 is present, the GD of H1/H5 binds to the nucleosome on the dyad as observed in the H1/H5-bound mononucleosome, while the C-terminal and N-terminal tails bind to each of the linker DNA, respectively. The binding of H1/H5 neutralizes the negative charges of linker DNA and reduces the repulsion between nucleosomes, which consequently alters the orientation and geometry of the linker DNA at entry/exit of the H1/5 bound-nucleosomes and results in a zig-zag conformation of the nucleosome array (Fig. 7b, Stage 2). Within the appropriate zig-zag trajectories of entry/exit DNAs upon the binding of H1/H5, the less torsionally constrained nucleosomes at the end of the array, i.e., N1 and N12 in the dodecanucleosome, approach to their adjacent nucleosomes, i.e., N3 and N10, in a face-to-face stack, which is mainly mediated by the interactions between the H2A-H2B α-helical bundles on the surfaces of adjacent nucleosomes (N1/N3 or N12/N10) (Fig. 7b, Stage 3). Once the first nucleosomal stack is initiated and stabilized (N1/N3 or N12/N10) from the end, the chromatin folding is synergistically and directionally propagated fast along the fiber. In this way, the nucleosome array compacts into two-start parallel nucleosome stacks with tetranuclesome as a repeating unit (Fig. 7b, Stage 4). Meanwhile, the asymmetric binding of H1/H5 C-tail and N-tail to the linker DNA confers a polarity to the nucleosome, making the adjacent nucleosomes within the tetranucleosomal units, e.g., N1/N3, or N2/N4, arranged in a way to adopt an opposite (head-to-head) polarity in relation to the H5 location, which facilitates the folding of the tetranucleosomal units and forms a tetranucleosome-on-a-string state. This opposite arrangement of the H5 polarity within the tetranucleosomal unit yields a different spatial distribution of CTD-bound linker DNA and arrangement of adjacent nucleosomes, which consequently results in the interactions between tetranucleosomal units, e.g., H5–H5 associations and H4-N tails–H2A-2B acidic patch interactions, to form a two-start chromatin fiber. Having established the intra-tetranucleosomal interactions, the chromatin fiber begins to twist due to the asymmetrical force imposed by asymmetrical bindings and interactions, leading to the H5 binding mode switch from on-dyad to off-dyad and the formation of a two-start left-handed double-helical chromatin fiber twisted by tetranucleosomal units with a zig-zag configuration (Fig. 7b, Stage 5), as shown in our H5-chromatin fiber structure (Fig. 7c).

Although it remains controversial whether the 30-nm chromatin fiber exists in the nucleus, there is an increasing body of studies on in vivo chromatin structure including the recently published results supporting the assembly of chromatin structures in an overall similar form which we describe here.^73–75^ In this study, using a series of genetic, genomic, and biophysical approaches, we demonstrated that the tetranucleosomal folding unit plays important roles in regulating chromatin compaction and gene transcription, which is essential for cell growth and DNA damage response in yeast. For example, we found that the severe phenotype of H2B-R119G mutation can be rescued by the H2B-R119G/H2A-G23R double mutation, which restores the weakened intra-tetranucleosomal interaction in yeast (Fig. 4d, g, i). Interestingly, the H2A-G23R mutation alone shows little impact on the chromatin compaction and gene transcription. It may be attributed, at least in part, to the flexibility of the H2A N-terminus, which needs further study.

One important issue for our in vivo assays in yeast is whether the mutations on histone residues involved in the intra-tetranucleosomal interactions may affect the nucleosome structure and dynamics. First, to our knowledge, no functional post-transcriptional modifications (PTM) are reported for the histone residues mutated in this study in budding yeast. Second, according to the structure of nucleosomes assembled with yeast histones,^76^ most of the residues mutated in this study are located at the nucleosome surface at a long distance, indicating that they do not interact with each other on the same nucleosome. Some histone residues are reported to participate in the interactions with nucleosome remodelers or histone modification enzymes, while our rescue experiments strongly support that these mutations on histone residues mainly affect the tetranucleosomal folding unit, rather than the mononucleosome. Briefly, our results fill in the gap between the in vitro structural studies of the chromatin fiber and its biological relevance in vivo.

Although the multiple-level asymmetries of the nucleosome and the polarity of the chromatin fiber were revealed in the cryo-EM structure, their biological consequence in vivo remains largely unknown. Since apparent different structural conformations were observed in the core histone tails, which are extensively modified by various PTMs, it is reasonable to hypothesize that the histones could be modified asymmetrically in the context of chromatin fiber. In line with this, several recent genomic, biochemical, and biophysical studies have shown that nucleosomes in biological contexts are often asymmetric with histone compositions (exchange of histone variants), PTMs, chromatin remodeling, nucleosome positioning, and DNA flexibility.^77–80^ Taken together, our structure revealed that the multiple asymmetries of nucleosome structure confer a polarity to the chromatin fiber, which may play an important role in the asymmetrical reading of genetic information at transcription and unidirectional establishment of transcriptionally silent heterochromatin.^81^

## Materials and methods

### Proteins and DNA

Recombinant WT *X. laevis* and human histones (H2A, H2B, H3, and H4) were expressed in *Escherichia coli* BL21 (DE3) cells and purified as previously described.^82^ Histone H5 was expressed in *E. coli* Rosetta (DE3) cells using a pET42b vector with codon-optimized coding sequences of H5 and purified through a modified procedure established previously.^83^ Briefly, the cultured bacterial cells were harvested by centrifugation, resuspended in lysis buffer (50 mM Tris-HCl, pH 8.0, 50 mM EDTA, 1% Triton X-100, 1 mM PMSF), and lysed by high-pressure homogenizer. The insoluble material was collected and washed three times in wash buffer (50 mM Tris-HCl, pH 8.0, 1 mM EDTA, 100 mM NaCl, 1 mM PMSF) and dissolved in elution buffer (50 mM Tris-HCl, pH 8.0, 50 mM EDTA, 1 M NaCl, 1 mM PMSF) for 12 h on a stirrer at 4 °C. After centrifugation at 4 °C for 30 min, the supernatant was collected and dialyzed with buffer A (50 mM Tris, pH 8.8, 0.5 M NaCl, 1 mM PMSF) for 4 h. The insoluble material was removed by centrifugation, and the supernatant was collected and loaded on a Heparin ion exchange chromatography (GE Healthcare) for further purification. The H5 protein was eluted with buffer B (50 mM Tris, pH 8.8, 1.5 M NaCl, 1 mM PMSF) at 4 °C in a linear gradient using the AKTA pure system (GE Healthcare). The fractions containing H5 were pooled and dialyzed against BC-100 buffer (20 mM Tris, pH 7.5, 100 mM NaCl, 0.1 mM EDTA, 15% glycerol, 1 mM PMSF, 5 mM β-mercaptoethanol), then concentrated and stored at –80 °C.

The GD of H5, GH5_22–95_, was expressed in *E. coli* BL21 (DE3) cells and purified using Ni-NTA beads (Qiagen, CA). The His-tag was removed by thrombin (Roche, CA) and the protein was further purified using cation exchange (HiTrap SP, GE Life Sciences). The fractions containing GH5_22–95_ were concentrated and stored at –80 °C.

The DNA with 12× 177 bp_601 DNA tandem repeats of the 601 sequence for H5-chromatin reconstitution and the 167 bp DNA with the Widom “601” DNA in the middle for H5-NCP or GH5-NCP reconstitution were cloned and purified as described previously.^84^

### Reconstitutions of histone octamers, nucleosomes, and chromatin fibers

The histone octamers were reconstituted as previously described.^84^ Briefly, the H2A, H2B, H3, and H4 were mixed at equimolar amounts in unfolding buffer (7 M guanidinium HCl, 20 mM Tris-HCl, pH 7.5, 5 mM β-mercaptoethanol), and then dialyzed in refolding buffer (2 M NaCl, 10 mM Tris-HCl, pH 7.5, 1 mM EDTA, 5 mM β-mercaptoethanol). The resulting histone octamers were purified through a size exclusion chromatography column (Superdex 200, GE Healthcare), and the peak fractions were collected and stored.

Nucleosome arrays and chromatin fibers were assembled using the salt dialysis method as previously described.^84^ The histone octamers and 12× 177 bp_601 DNA were mixed at equimolar amounts in TEN buffer (10 mM Tris-HCl, pH 8.0, 1 mM EDTA, 2 M NaCl), and was continuously diluted by slowly adding TE buffer (10 mM Tris HCl, pH 8.0, 1 mM EDTA) over 16 h at 4 °C to the concentration of NaCl from 2 M to 0.6 M. Then, the H5 protein, an equal molar amount relative to mono-nucleosomes, was added and further dialyzed against TE buffer containing 0.6 M NaCl for 3 h, followed by a final dialysis step in HE buffer (10 mM HEPES, pH 8.0, 0.1 mM EDTA) for 4 h. Quality of the chromatin sample was monitored by negative staining electron microscopy and AUC analyses. The well-prepared H5-chromatin sample was chemically fixed with 0.2% GA and used for cryo-EM sample preparation.

The H5-chromatosomes (H5-NCP) were assembled using the same protocol as the above-described H5-chromatin fibers. Briefly, the histone octamers and 167 bp_601 DNA were mixed at equimolar amounts in TEN buffer with 2 M NaCl, and the concentration of NaCl was slowly diluted from 2 M to 0.6 M to assemble the mono-nucleosome (NCP). Then, the H5 protein was added at an equal molar amount relative to mono-nucleosome and further dialyzed in buffer with 0.6 M NaCl for 3 h, followed by a final dialysis step in HE buffer (10 mM HEPES, pH 8.0, 0.1 mM EDTA) for 4 h. The resulting chromatosome (H5-NCP) in HE buffer was chemically fixed and concentrated for cryo-EM sample preparation.

For GH5-NCP, the NCP in 0.6 M NaCl was dialyzed in HE buffer for 4 h. The GH5 was then added with an equal ratio to NCP and incubated at ice for 2 h. The sample was then chemically fixed and concentrated for cryo-EM sample preparation.

### Sedimentation velocity-AUC

Chromatin samples containing linker histone H5 were diluted with HE buffer to 400 μL. Sedimentation experiments were performed on a Beckman Coulter ProteomeLab XL-I with a 60Ti rotor. The initial absorbance of the samples at 260 nm was adjusted to 0.4–0.8. Samples were pre-equilibrated for 1.5 h at 20 °C under a vacuum condition. The absorbance at 260 nm was measured during sedimentation at 20,000× *g* with standard 2-channel centerpiece cells. Data were analyzed using enhanced van Holde–Weischet analysis and the Ultrascan II/III software. The S_20,w_ values (sedimentation coefficient corrected for water at 20 °C) were calculated with a partial specific volume of 0.622 mL/g for the chromatin sample, and the buffer density and viscosity were adjusted. The average sedimentation Save coefficients were determined at the boundary midpoint.

### Single-molecule magnetic tweezer analysis

The tetra-nucleosome stretching experiments were performed by single-molecule magnetic tweezers.^22,85^ Briefly, the two DNA ends of reconstituted tetra-nucleosome were anchored by digoxigenin and anti-digoxigenin ligation to a glass coverslip and by biotin and streptavidin ligation to a 2.8-μm Dynabeads (M280, Invitrogen, Norway), respectively. The applied tensions on the super-paramagnetic beads derived from the strong magnetic field gradient of the two anti-parallel NdFeB magnets with a distance of 0.5 mm, which were controlled to manipulate the Dynabeads and thus stretch the tetra-nucleosome samples. The flow cell was rinsed with 200 μL HE buffer. The bead image was projected onto the CCD camera (MC1362, Mikrotron) through an inverted microscope objective (UPLXAPO60XO, NA 1.42, Olympus). The real-time position (*x*, *y, z*) of the bead was recorded by comparing the diffraction pattern with the calibration images at various distances from the focal point of the objective based on the quadrant-interpolation (QI) algorithm.^86^ About 100 samples can be traced simultaneously in each measurement. In the measurement, the magnets were moved near to the samples at a rate of 10 μm/s, during which the extension change due to the unfolding of the tetra-nucleosome was recorded. All measurements were carried out at 25 °C. To confirm the force in the measurement, the force calculation for a 10,000-bp DNA tethered between the bead (M280) and the coverslip was carried out before these measurements. At each magnet position, the *y*-position of the bead was recorded at 500 Hz for 5 min and the corresponding force was calibrated by power-spectral-density analysis.^87^ The force measurements were repeated for more than 10 independent DNA tethers. The relationship between force and magnet position was fitted well with a double exponential function.

### Cryo-EM

The reconstituted H5-chromatin fibers and chromatosomes (H5-NCP or GH5-NCP) were chemically fixed with 0.2% GA. Aliquots of 3.5 μL sample were applied onto glow-discharged Quantifoil R2/1 300 mesh holey carbon grids, incubated for 1 min at 4 °C and 100% humidity, blotted for 3 s with a filter paper and then plunged into liquid ethane using an FEI Vitrobot Mark IV (ThermoFisher Scientific).

For the H5-chromatin sample, cryo-EM image stacks were collected in a Titan Krios microscope (ThermoFisher Scientific) operating at 300 kV acceleration voltage and equipped with a Falcon II direct electron detector (ThermoFisher Scientific) at a magnification of 47,000× with a pixel size of 1.76 Å. The images were recorded using SerialEM software^88^ at a total dose of about 60 e^−^/Å^2^ (30 frames) with a defocus range from –2.0 μm to –3.0 μm.

For the H5-NCP sample, cryo-EM image stacks were collected in a Titan Krios microscope (ThermoFisher Scientific) operated at 300 kV acceleration voltage and equipped with a Volta phase plate (VPP) (ThermoFisher Scientific) and a Gatan K2 Summit direct electron detector with Quantum energy filter (Gatan), at the super-resolution mode with a nominal magnification of 130,000×, corresponding to a calibrated super-resolution pixel size of 1.04 Å. The images were recorded using SerialEM software^88^ at a total dose of about 60 e^−^/Å^2^ (32 frames) with a defocus range from –0.5 μm to –1 μm.

For the GH5-NCP sample, cryo-EM image stacks were collected in the same microscope and pixel size with H5-NCP, but without using the Volta phase plate. The images were recorded at a total dose of about 60 e^−^/Å^2^ (32 frames) with a defocus range from –1.5 μm to –2 μm.

### Cryo-EM image processing and 3D structure reconstruction

All of the cryo-EM image processing and 3D structure reconstructions were performed in Relion 3.0, 3.1, and/or cryoSPARC2.0.^89–92^ The beam-induced motion correction and dose-weighting were carried out using MotionCor2^93^ in Relion 3.0.^90,91,94^ CTF parameters were estimated using either Gctf^95^ for H5-NCP VPP dataset or CTFFIND4.1^96^ for H5-chromatin and GH5-NCP datasets.

For H5-chromatin, 2851 cryo-EM micrographs with dose-weighting sums were acquired. 56 of them were discarded with bad behavior of CTF Thong rings. 43,402 raw particles were manually picked from the remaining 2795 micrographs. After one round of 2D classification in Relion3.0, particles in the classes with smeared or broken features were abandoned and the remaining 16,070 particles were subjected to 3D classification. Starting with an initial model generated from a low-pass filtered cryo-EM map (EMDB ID: 2600)^21^ with a 45 Å cut-off, three 3D classes with obvious 12-mer nucleosomes were combined for further 3D auto-refinement which resulted in a 4.1 Å density map. After three rounds of iterative refinements with correction of optical aberration, anisotropic magnification, per-particle defocus, and Bayesian particle polishing, a 3.6 Å map was obtained, which was then sharpened by applying a negative B-factor of –92 Å^2^, and corrected for modulation transfer function (MTF) of Falcon II detector. The local resolution of the density map was estimated using the post-processing program in Relion.

For the dataset of H5-NCP, 1677 micrographs were collected in which 89 micrographs with unfitted phase shifts and aberrant CTF were discarded. Initially, about 1500 particles were visually boxed out to generate a 2D template for subsequent template-based auto-picking in Relion. Totally, 536,264 raw particles were picked and downsampled for 2D classification to remove junks, resulting in a subset of 408,540 particle images. With a starting reference from a low-pass filtered PDB model (ID: 2NQB), 3D classification was performed and one of the nine classes containing 46,427 particles with obvious density bridging entry and exit point of linker DNA was re-extracted for 3D auto-refinement. A map of 3.9 Å resolution was obtained after auto-refinement. Based on the auto-refined results, four rounds of iterative refinements with correction of global and local defocus values, Euler angles, phase shifts, and particle polishing were performed producing a density map of global resolution at 3.6 Å. Due to its intrinsic flexibility of NTD and CTD of histone H5, the density connecting both ends of two linker DNA is more noisy relative to other regions of NCP. In order to achieve a map of H5 locally better resolved, an additional focused 3D classification without particle image alignment was conducted, with the density of histone octamer masked out. One class containing 33,753 particles showed distinct H5 density was exported for a final non-uniform refinement in cryoSPARC,^89^ resulting in a final reconstruction of 3.3 Å with a sharpening B-factor of –53.9 Å^2^.

For GH5-NCP dataset, a similar image-processing strategy was applied. 276,401 particles were auto-picked using convolutional neural networks (CNN)-based Topaz^97^ and at last, a subset of 45,705 particles was used to produce a map at 3.6 Å resolution with a B-factor of –114 Å^2^.

The reported resolution for all reconstructions are based on the gold-standard Fourier shell correlation (FSC) at 0.143 criterion,^94,98^ and high-resolution noise substitution was applied to correct for the effects of soft masks during FSC calculation.^99^

For details of image processing for all the above three samples, see Supplementary information, Figs. S2, S5, and Table S4.

### Model building and refinement

Cryo-EM map segmentation, interpretation, and atomic model building were performed with UCSF Chimera^100^ and Coot,^101^ respectively. The structural model was first built by fitting the crystal structures of the NCP (PDB: 1KX5) and H5 GD domain (PDB: 1HST) to the 3.6 Å EM density map using UCSF Chimera. The model of H5 NTD and CTD domains was built de novo in Coot according to the density map. The model was further refined using the Phenix. real_space_refine in Phenix^102^ with secondary structure constraints enforced. The refined model was then rebuilt manually in Coot to improve the atomic model fitting, refine the geometries, and remove the atom clashes. The geometry parameters of the final models were validated using MolProbity.^103^ The refinements were performed iteratively until no further improvements were observed. The parameters for cryo-EM data collection and statistics for atomic model building and refinement are listed in Supplementary information, Table S4.

All figures were prepared with UCSF Chimera,^100^ UCSF ChimeraX,^104^ Pymol (https://www.pymol.org), and ESPript 3.0.^105^ The intra- and inter-tetranuclesomeal unit interactions in the H5-chromatin structure were analyzed using PDBePISA.^68^

### Yeast strain

All *S. cerevisiae* strains were YPH500 derivatives, as detailed in Supplementary information, Table S2. The gene deletion strains were generated by the method reported.^69,106,107^ In brief, we first got the strain with the original two *H2A* and *H2B* genes deleted by replacing the *HTA1-HTB1* locus with *TRP1*, reintroducing *HTA1-HTB1* genes with endogenous promoter, terminator, and open reading frame on a *URA3* containing pRS316 plasmid, and then replacing the *HTA2-HTB2* locus with *HIS3*. To get strains with only mutant histone genes, we transformed another CEN plasmid pRS315 which carries the endogenous *HTA1-HTB1* gene locus but mutated fragment. Shuffle assays for fully mutant histones were done as ref. ^3^ by culturing the strain on a SC + 5-FOA plate and incubated at 30 °C for up to 3 days. The single colonies on the SC-Leu plates were picked and streaked. Spot assays (as indicated) were diluted 10-fold from fresh cultures (A_600_ of ~1.0) and spotted on the plates with/without stress.

### RNA-seq

Each strain was subjected to RNA-seq analysis with at least three biological replicates. Briefly, each biological replicate was grown at 30 °C in 15 mL YPD to an A_600_ between 0.6 and 0.8 from an initial starting A_600_ of 0.1. Cultures were spun down, and the harvested cells with DEPC water were washed. Sangon Yeast Total RNA Isolation Kit (B518627-0100) was used to extract RNA. RNA libraries were prepared according to the Illumina TruSeq protocol and sequenced using a HiSeq 2000 system at Berry Genomics.

### Micro-C

Micro-C library preparation was performed as previously described.^24,108^ In short, yeast cells (100 mL, OD_600_) were fixed with 3% formaldehyde (Sigma-Aldrich, 252549) for 10 min at 30 °C and then quenched with 0.75 M Tris-HCl (pH 7.5). Quenched cells were pelleted, washed, and resuspended in 120 mL Buffer Z (1 M sorbitol, 50 mM Tris, pH 7.4, and 10 mM β-mercaptoethanol) and spheroplasted with Zymolyase for 45 min at 30 °C. After twice wash with cold PBS, spheroplasts were resuspended in 5 mL of 3 mM EGS solution and crosslinked for 45 min at RT with rotation, and quenched by additional Tris-HCl (pH 7.5) to 0.75 M. Crosslinked spheroplasts were pelleted, washed in cold PBS, and pelleted again prior to flash freezing. The spheroplasts were split by MNase-treatment to yield > 90% mononucleosomes, followed by end repair and labeling, proximity ligation, di-nucleosomal DNA purification, and library preparation as described.^24^ Di-nucleosomal DNA was purified following the gel extraction step and the library was constructed with NEB Next Ultra DNA library Prep kit (E7645L) for Illumina. The PCR product was purified with 1 volume AMpure beads. The libraries were sequenced on Nova-seq6000 with paired-end 150 bp (Illumina). Each strain was completed in at least two biological replicates.

### RNA-seq and gene expression analysis

The pair-end RNA-seq reads were mapped to the *S. cerevisiae* SacCer3, gene annotation model using Subread^109^ with the default parameters, and the only uniquely mapped reads were used for further analysis. The differential expression between samples was performed with featureCounts^110^ and R package DESeq2.^111^ Gene enrichment for one gene group was analyzed by the Metascape.^112^

### Micro-C analysis

Micro-C datasets were processed with the HiC-Pro analysis pipeline.^113^ Briefly, reads were mapped to the *S. cerevisiae* reference assembly sacCer3 using Bowtie2 with “very sensitive” mode. Pairs with multiple hits, low MAPQ, singleton, dangling end, self-circle, and PCR duplicates were removed. Output files containing all valid pairs were used in downstream analysis. Analysis was completed separately for each orientation of ligation pairs (“IN-IN”, “OUT-OUT” and “IN-OUT”, which includes “IN-OUT” and “OUT-IN” pairs). “IN-OUT” contacts are shown in all figures. The cumulative sum of the contact probability within a genomic distance range is reported as the cumulative contact probability.

To test the enrichment of the detected loops, we performed the aggregated peak analysis (APA) using juicer tools^114^ with default settings. In brief, fold change values quantifying enrichment of contact frequency compared to local background were calculated. Then we tested whether the number of the observed contacts is significantly enriched. The APA score was also calculated by the Juicer APA command as the ratio of micro-C contacts at the central pixel vs the mean micro-C contacts of the lower-left pixels.

## Supplementary information

Supplemental material

Video S1

Video S2

Video S3

## Data Availability

The cryo-EM maps for the 12× 177 bp H5-chromatin fibers, H5- and GH5-bound 167 bp NCPs were deposited into the Electron Microscopy Data Bank with the accession codes EMD-38407, EMD-37637 and EMD-37638, respectively. The coordinates of 12× 177 bp H5-chromatin fiber were deposited to the Protein Data Bank with accession code PDB- 8XJV. RNA-seq and micro-C data have been deposited in the National Genomics Data Center, China National Center for Bioinformation/Beijing Institute of Genomics, Chinese Academy of Sciences (CRA011579) that are publicly accessible at https://ngdc.cncb.ac.cn/gsa. Further information, resources, and reagents are available from P.Z. and G.L. upon reasonable request.
