# Supplementary material for: Structural basis for linker histone H5–nucleosome binding and chromatin fiber compaction
Source: Cell Res. 2024 Aug 5;34(10):707–24. doi: 10.1038/s41422-024-01009-z (PMC11442585; doi:10.1038/s41422-024-01009-z)
Supplement: Supplementary file 1 — Supplemental material [file 41422_2024_1009_MOESM1_ESM.pdf]

Supplementary Information for

Structural basis for the linker histone H5-nucleosome binding and chromatin compaction

Wenyan Li[1,2]†, Jie Hu[1,2]†, Feng Song[3,4]†, Juan Yu[1]†, Xin Peng[1,2], Shuming Zhang[5], Lin Wang[1,2], Mingli Hu[1,2], Jia-Cheng Liu[6], Yu Wei[1,2], Xue Xiao[1,7], Yan Li[1], Dongyu Li[1,2], Hui Wang[1,2], Bing-Rui Zhou[8], Linchang Dai[1,2], Zongjun Mou[1,2], Min Zhou[1], Haonan Zhang[1,2], Zheng Zhou[1,2], Huidong Zhang[9], Yawen Bai[8], Jin-Qiu Zhou[6], Wei Li[1,7], Guohong Li[1,2,3*] and Ping Zhu[1,2*]

†These authors contributed equally to this work.

[*]Corresponding author. E-mail: zhup@ibp.ac.cn (P.Z.); liguohong@whu.edu.cn (G.L.)

This document includes:

Supplementary Figures (Fig. S1 - Fig. S12)

Supplementary Tables (Table S1 - Table S4)

Supplementary Video legends (Video S1 - Video S3)

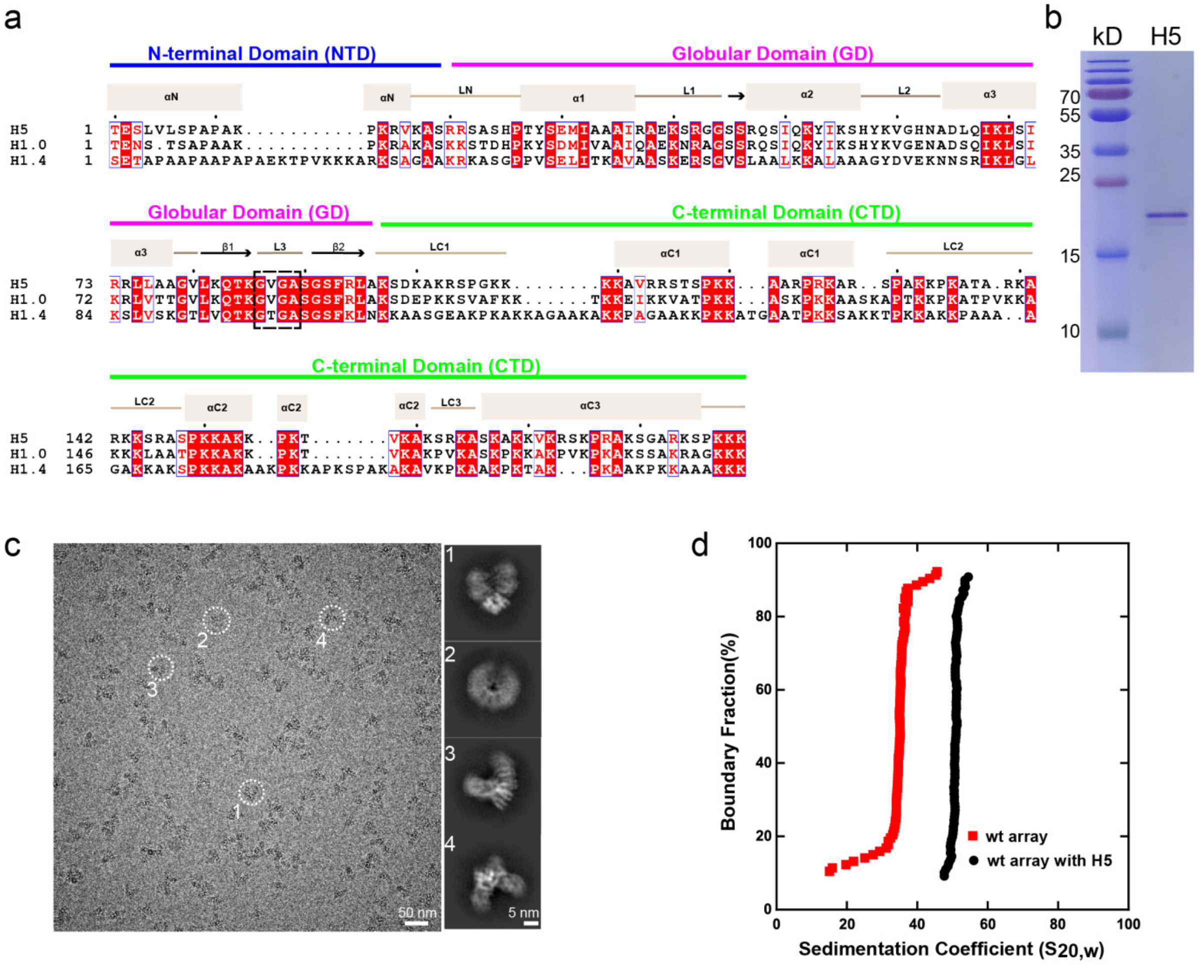

Supplementary information, Fig. S1 Structural analysis of the H5-chromatin fiber reconstituted with 12×177_601 DNA. a. Amino acid (aa) sequence of H5 aligned with human H1.0 and H1.4. The highly conserved residues are highlighted in red backgrounds. The three structural domain regions, i.e., NTD, GD, and CTD, of H5 and the secondary structure elements revealed from the H5-chromatin structure are depicted and labeled above the aa sequence. **b.** SDS–PAGE of the purified histone H5 used in this study. **c.** A representative cryo-EM micrograph of the H5-chromatin particles. The insets show typical 2D class averages corresponding to the selected raw particle labeled with the same number. **d.** The sedimentation velocities of the nucleosomal arrays and the H5-chromatin fiber by analytical ultracentrifugation (AUC) analysis.

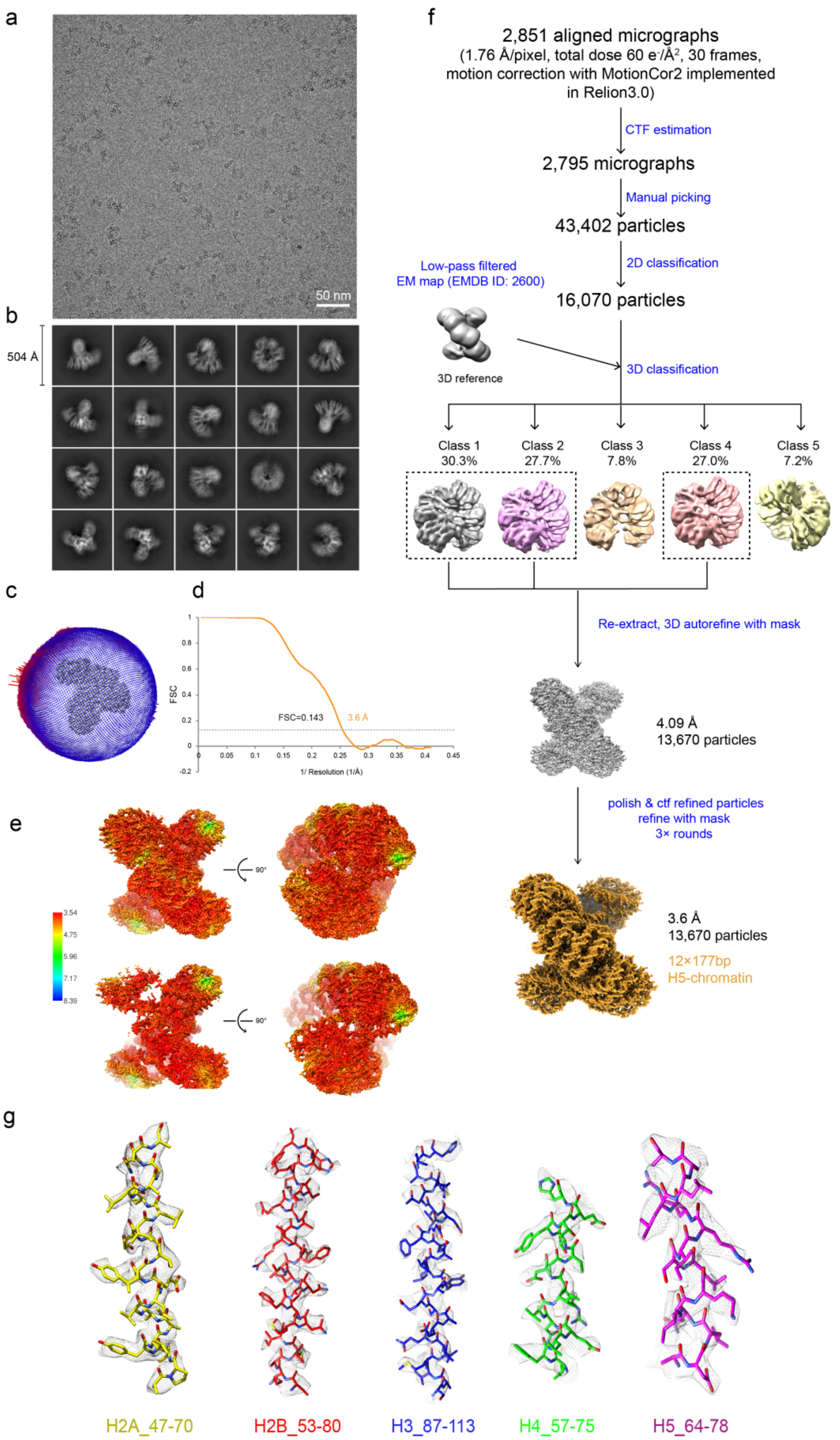

Supplementary information, Fig. S2 The procedure of cryo-EM 3-D structure reconstruction of the H5-chromatin fiber. a. A representative cryo-EM micrograph of the reconstituted H5-chromatin fiber. **b.** Two-dimensional

class averages show characteristic projection views of the H5-chromatin particles. **c.** Angular distribution of the particle projections of the H5-chromatin fiber. d. The Fourier Shell Correlation (FSC) curve shows a reconstruction resolution of 3.6Å at 0.143 cutoff. **e.** Cryo-EM density maps of the H5-chromatin fiber colored on the basis of the local resolution. **f.** Flow chart of 3-D reconstruction process of the H5-chromatin fiber. **g.** Segmented densities of H2A, H2B, H3, H4 and H5 (mesh, in gray) from the reconstructed cryo-EM density map of H5-chromatin fiber fitted with the corresponding atomic model (sticks, in color).

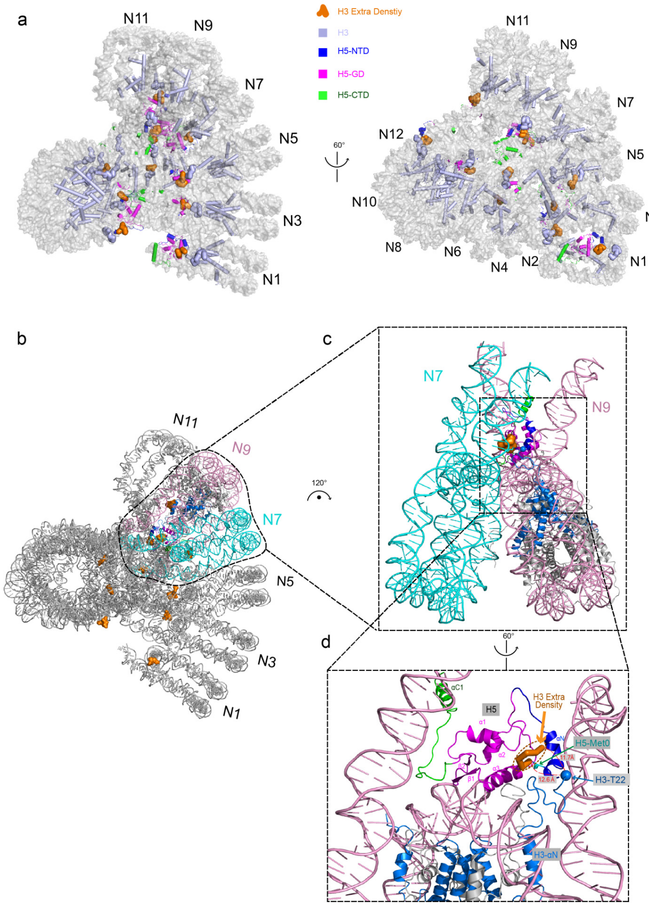

Supplementary information, Fig. S3 The presumptive H3-NTD densities in the cryo-EM structure of H5-chromatin fiber. a. The reconstructed 3-D cryo-EM density map (grey) of H5-chromatin fiber viewed from two different angles show some unassigned extra densities (orange) likely contributed by the H3-NTD. Histone H3 (light blue), H5-NTD (blue), H5-GD (magenta) and H5-CTD (green), are shown to display their relative locations to the extra densities. **b.** Distribution and locations of the presumptive H3-NTD extra densities in the H5-chromatin shown in carton representation. For clarity, only the histones of N9 are shown. **c.** Enlarged-view of the dashed part in (b) shows the H3-NTD extra density of N9. The DNA of N7 (cyan) and N9 (light pink) are shown to display the relative positions. **d.** Enlarged view of the dashed box in (c) exhibiting the details of H3-NTD extra density in N9 shown with H5 (NTD:blue, GD:magenta, CTD:green), H3 (light blue) and other core histones (grey). The extra density is shown in carton with ~6 amino acids, which contacts with the 1st residue (H5-M0, light green stick) of H5-NTD and in ~12Å distance to the furthest resolved residue (H3-T22, light blue sphere) of H3-NTD.

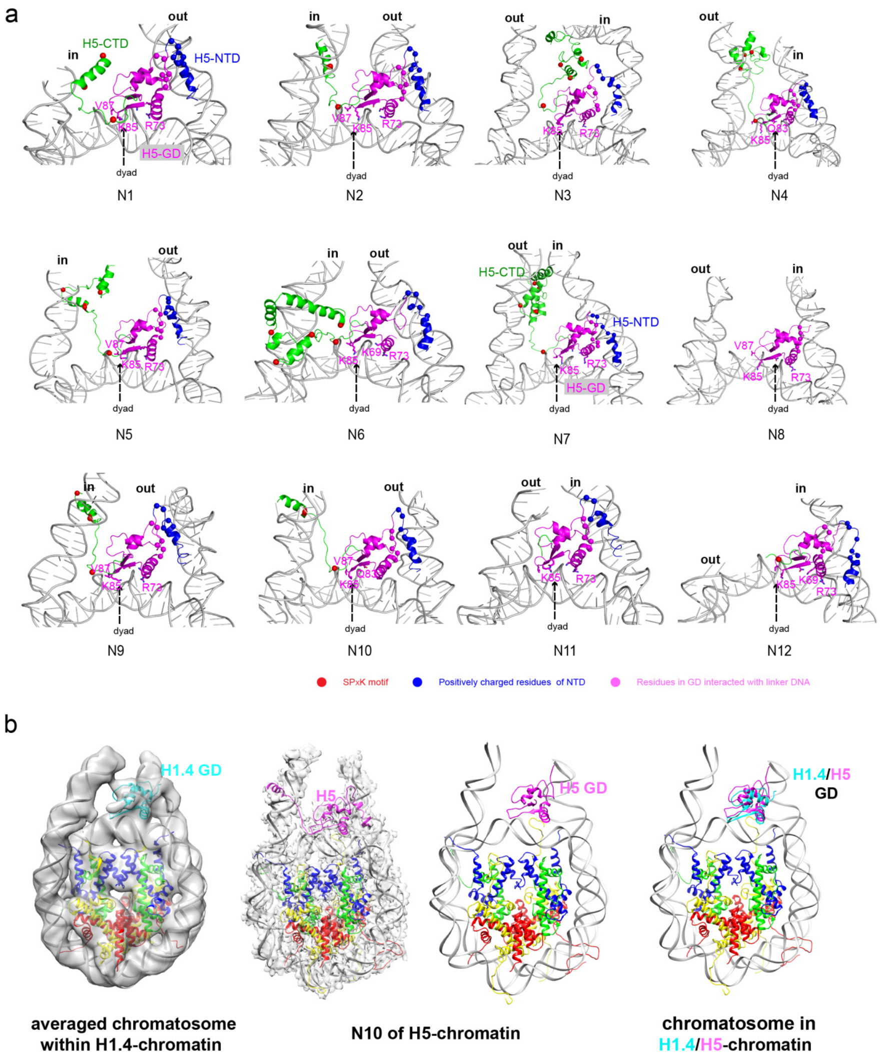

Supplementary information, Fig. S4 The asymmetric locations of linker histone in the H5-chromatin fiber. a. The location of H5 and its three distinct domains (NTD: blue, GD: magenta, CTD: green) in each chromatosome of the dodecanucleosome H5-chromatin fiber. The residues close to nucleosomal dyad axis and linker DNA in H5-GD are labeled and shown in sticks and magenta spheres, respectively. The positively charged amino acids in H5-NTD are shown in blue spheres and the serine residues in the SPxK motifs in H5-CTD are shown in red spheres. The dyad, entry and exit linker DNA of each chromatosome are indicated. **b.** Left: the asymmetric location of H1.4-GD (cyan) in an averaged H1.4-containing chromatosome density map (grey) from the H1.4-chromatin cryo-EM structures, fitted with the crystal structures of nucleosome core particle (pdb: 1AOI) [4] and Gallus histone H5 globular domain (GH5) (PDB:1HST) [21,60]. Middle: the asymmetric location of the H5-GD (magenta) in N10 of the H5-chromatin. Right: the H1.4/H5 chromatosomes with the nucleosome core aligned show the relative globular domain locations of linker histone H1.4 (cyan) and H5 (magenta).

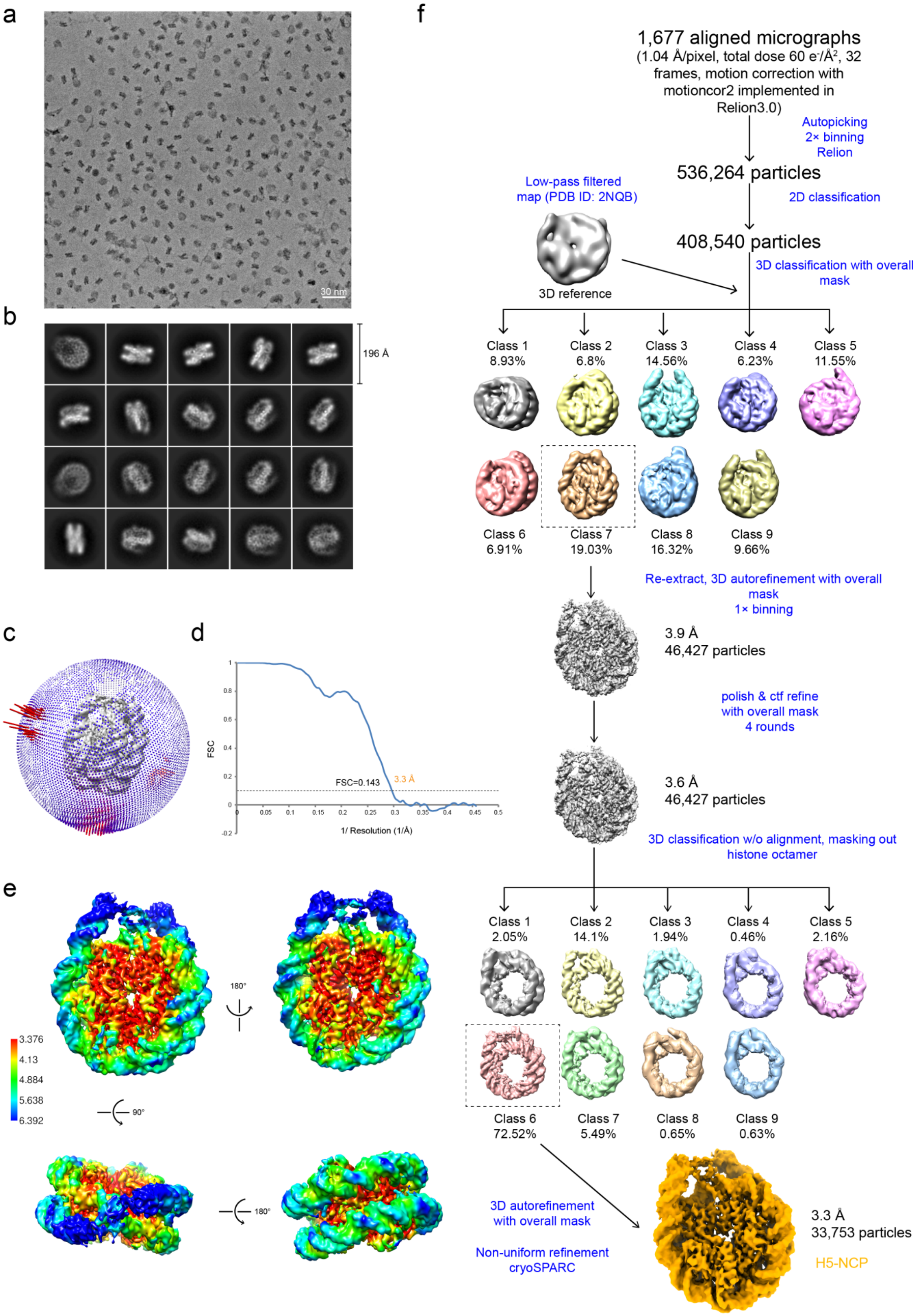
a
30 nm
b
196 Å
c
d
FSC
FSC=0.143
3.3 Å
1/ Resolution (1/Å)
e
3.376
4.13
4.884
5.638
6.392
180°
90°
180°
f
1,677 aligned micrographs
(1.04 Å/pixel, total dose 60 e-/Å2, 32 frames, motion correction with motioncor2 implemented in Relion3.0)
Autopicking
2× binning
Relion
536,264 particles
2D classification
Low-pass filtered map (PDB ID: 2NQB)
3D reference
408,540 particles
3D classification with overall mask
Class 1 8.93%
Class 2 6.8%
Class 3 14.56%
Class 4 6.23%
Class 5 11.55%
Class 6 6.91%
Class 7 19.03%
Class 8 16.32%
Class 9 9.66%
Re-extract, 3D autorefinement with overall mask
1× binning
3.9 Å
46,427 particles
polish & ctf refine with overall mask
4 rounds
3.6 Å
46,427 particles
3D classification w/o alignment, masking out histone octamer
Class 1 2.05%
Class 2 14.1%
Class 3 1.94%
Class 4 0.46%
Class 5 2.16%
Class 6 72.52%
Class 7 5.49%
Class 8 0.65%
Class 9 0.63%
3D autorefinement with overall mask
Non-uniform refinement cryoSPARC
3.3 Å
33,753 particles
H5-NCP

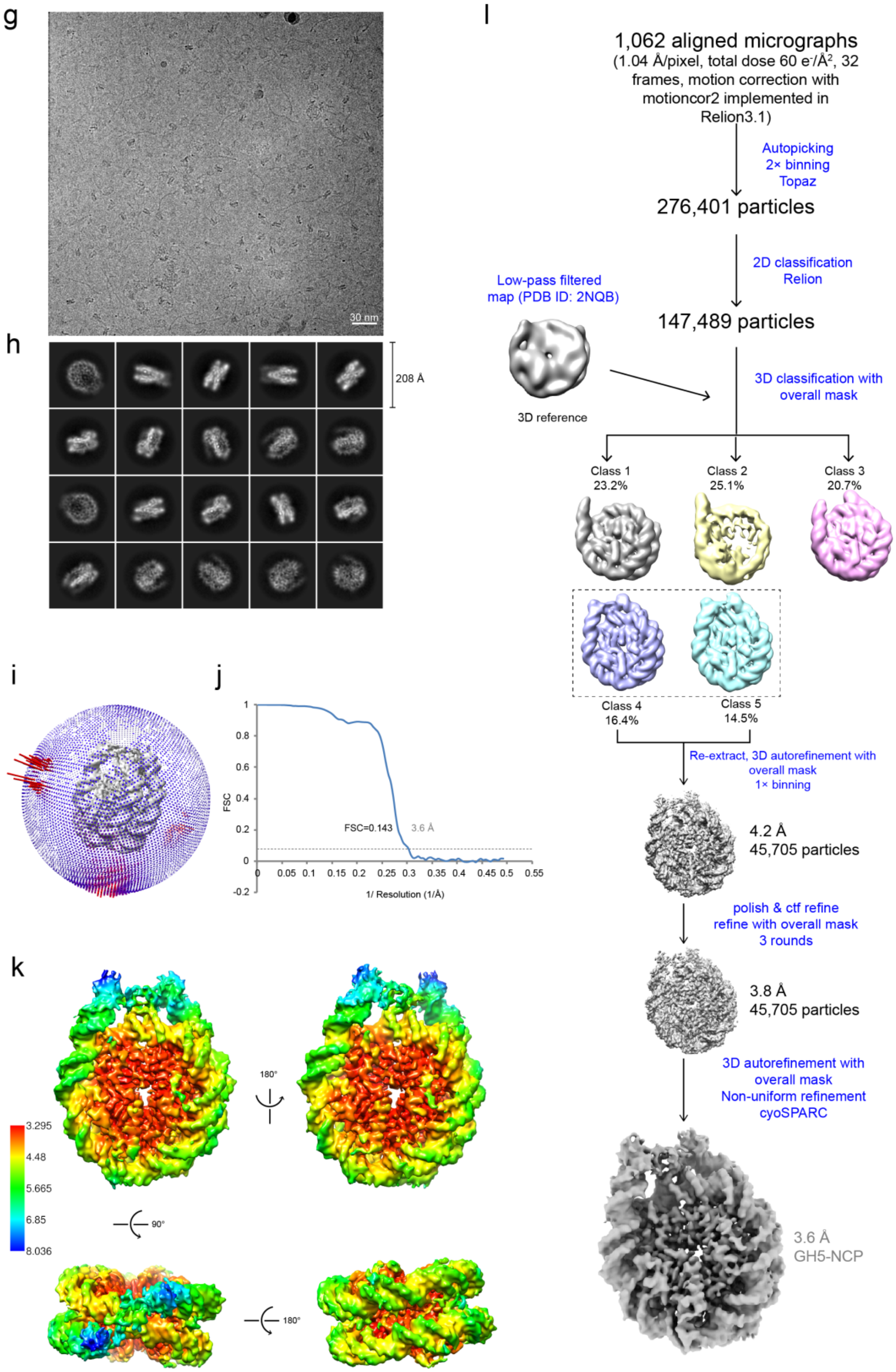
g
30 nm
h
208 Å
i
j
FSC
FSC=0.143
3.6 Å
1/ Resolution (1/Å)
k
3.295
4.48
5.665
6.85
8.036
180°
90°
180°
l
1,062 aligned micrographs
(1.04 Å/pixel, total dose 60 e-/Å², 32 frames, motion correction with motioncor2 implemented in Relion3.1)
Autopicking
2× binning
Topaz
276,401 particles
2D classification
Relion
Low-pass filtered map (PDB ID: 2NQB)
147,489 particles
3D reference
3D classification with overall mask
Class 1
23.2%
Class 2
25.1%
Class 3
20.7%
Class 4
16.4%
Class 5
14.5%
Re-extract, 3D autorefinement with overall mask
1× binning
4.2 Å
45,705 particles
polish & ctf refine
refine with overall mask
3 rounds
3.8 Å
45,705 particles
3D autorefinement with overall mask
Non-uniform refinement
cyoSPARC
3.6 Å
GH5-NCP

Supplementary information, Fig. S5 Cryo-EM structural analysis of the chromatosome with H5 or H5 GD. a. A representative raw cryo-EM micrograph of the H5-chromatomoe (H5-NCP complex). **b.** Two-dimensional class averages show characteristic projection views of the H5-NCP particles. **c.** Orientational distribution of the H5-NCP particle projections. **d.** The FSC curve of the H5-NCP shows a 3.3Å reconstruction resolution at 0.143 cutoff. **e.** EM density map of the H5-NCP viewed from different angles and colored by local resolution estimation. **f.** Flow chart of cryo-EM data processing of the H5-NCP. **g.** A representative raw cryo-EM micrograph of the GH5 chromatomoe (GH5-NCP complex). h**.** Two-dimensional class averages show characteristic projection views of the GH5-NCP particles. **i.** Orientational distribution of GH5-NCP particle projections. **j.** The FSC curve of the GH5-NCP shows a 3.6 Å reconstruction resolution at 0.143 cutoff. **k.** EM density map of the GH5-NCP viewed from different angles and colored by local resolution estimation. **l.** Flow chart of cryo-EM data processing of the GH5-NCP.

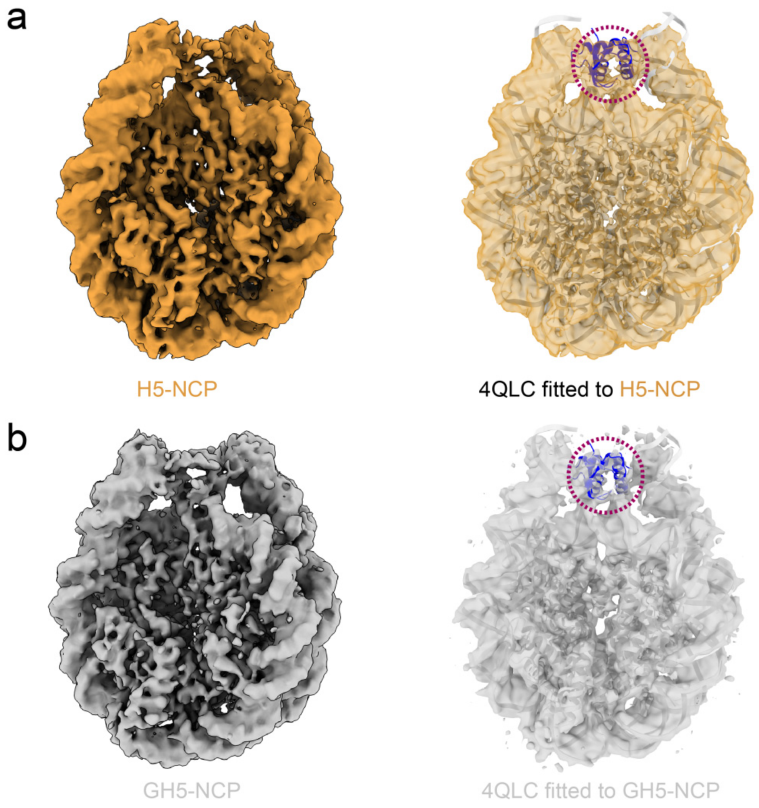

Supplementary information, Fig. S6 Cryo-EM maps of the H5-NCP and GH5-NCP. a. The reconstructed cryo-EM density map of H5-chromatosome (H5-NCP) (left) and the H5-NCP density map with fitted crystal structure of GH5-chromatosome (pdb:4QLC)[39]. The circle highlights the density corresponding to GH5 in the structure. **b.** The reconstructed cryo-EM density map of GH5-chromatosome (GH5-NCP) (left) and the GH5-NCP density map fitted with the crystal structure of GH5-chromatosome (pdb:4QLC)[39]. The circle highlights the GH5 region in the structure.

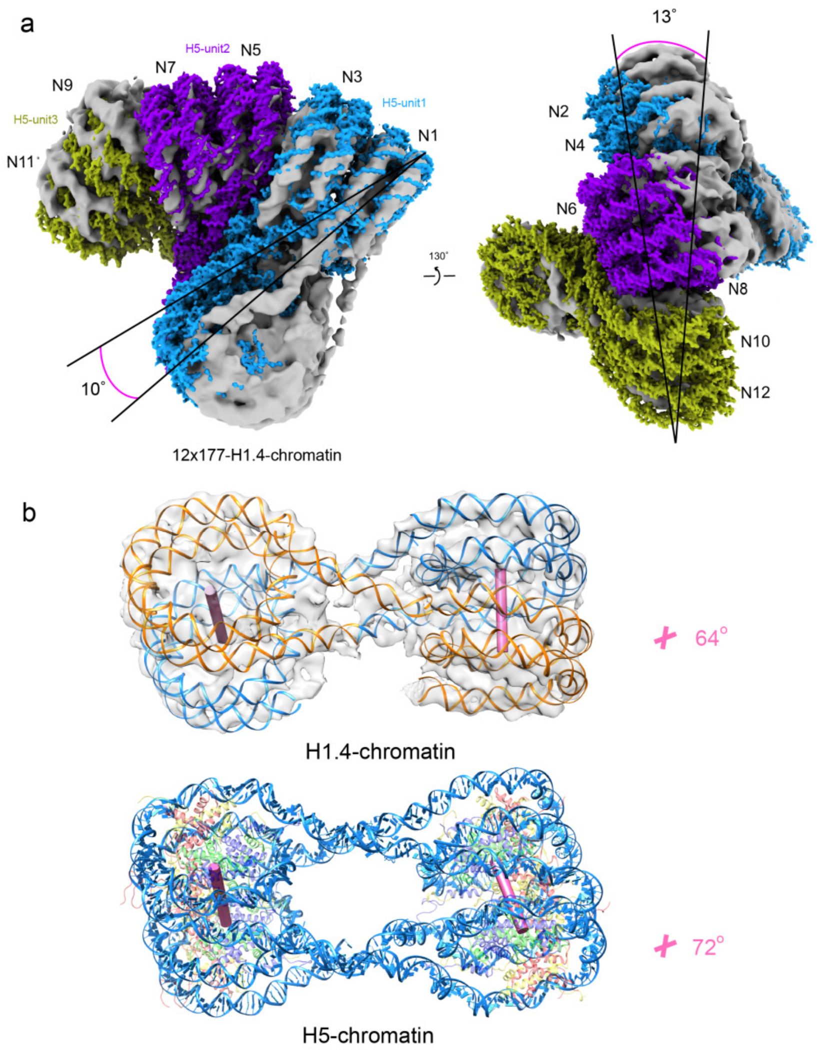

Supplementary information, Fig. S7 Structural comparison between the H1.4- and H5-chromatin fibers. a. Structural comparison of the 3D cryo-EM density map of 12×177 bp H5-chromatin fiber (color) with that of 12×177 bp H1.4-chromatin fiber (EMDB-2600, gray) [22]. The overall architectures of H5-chromatin and H1.4-chromatin fibers are viewed from two angles, which show a slightly more twisted conformation of H5-chromatin fiber than that of H1.4-chromatin as indicated by the angles. b. Top: The segmented density map of a tetranucleosomal unit in the H1.4-chromatin fiber shown with the fitted DNA in ribbon. Bottom: The structure of a tetranucleosomal unit in the H5-chromatin fiber. The axes of the two-nucleosome stacks within the tetranucleosomal unit are shown in pink and the twisted angles between two stacks axes of H1.4/H5-chromatin are labeled respectively.

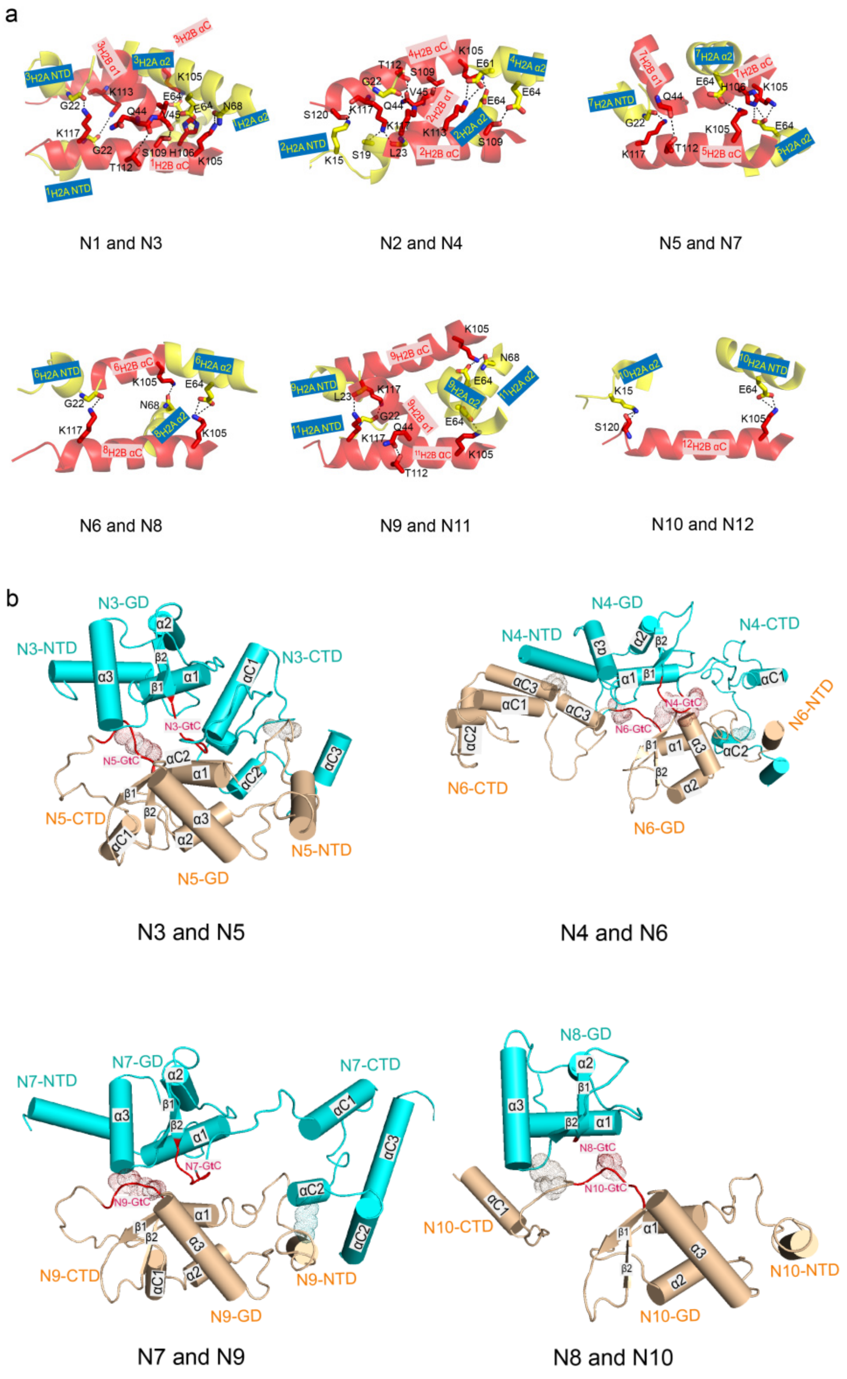

Supplementary information, Fig. S8 Nucleosomal interactions within tetranucleosomal unit in the H5-chromatin fiber. a. Six pairs of nucleosome-nucleosome interactions engaged by H2A-H2B and H2B-H2B within the tetranucleosomal units are shown. H2A and H2B are colored in yellow and red, respectively. The key residues involved in the interactions are shown in sticks and labeled. See also Supplementary information, Table 1. **b.** Four pairs of the H5-H5 interactions between adjacent tetranucleosomal units in the H5-chromatin fiber are shown. The meshed dots show the residues region involved in the interactions and the red colored sections show the GtC loop.

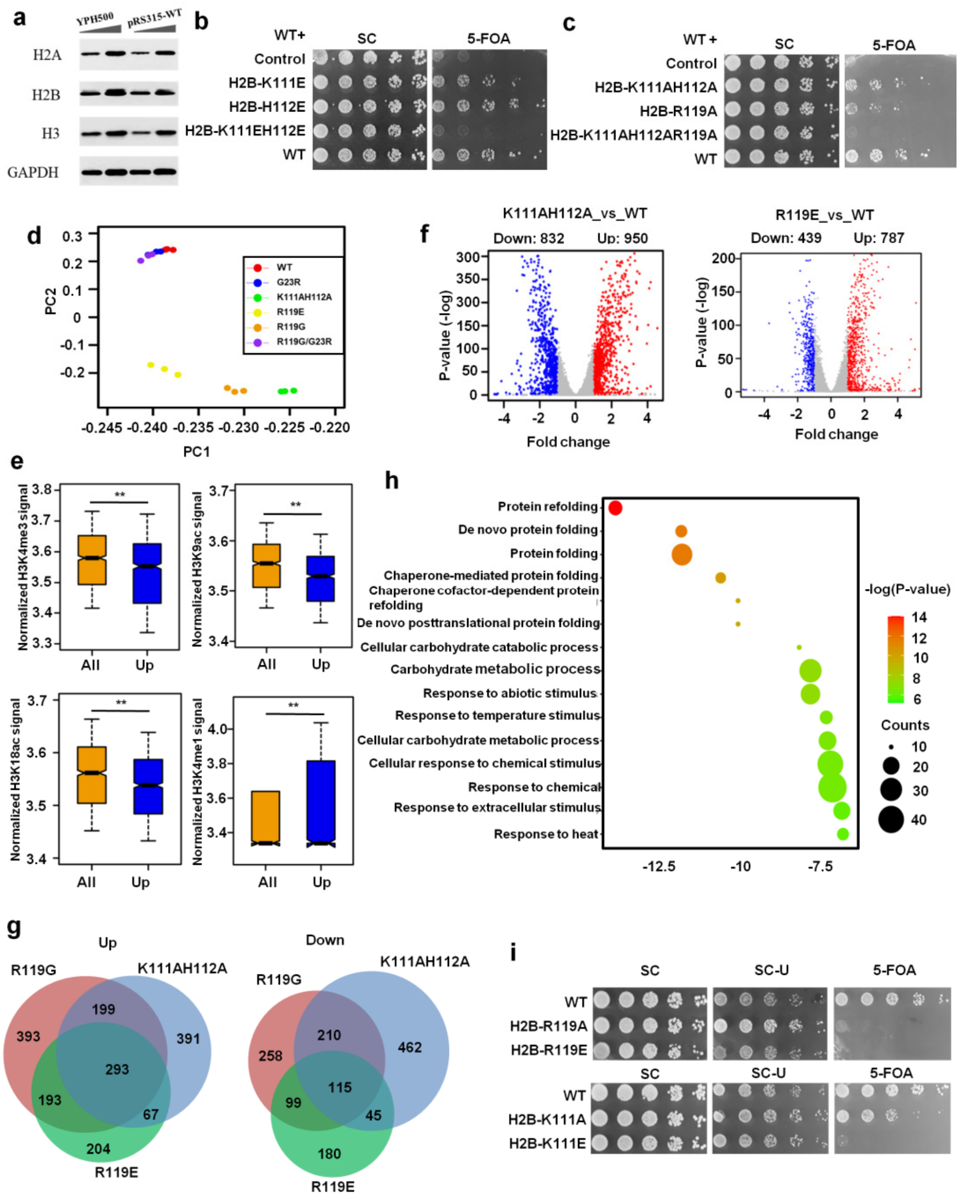

Supplementary information, Fig. S9 Change of viability and gene expression in mutants with destabilized tetranucleosomes. **a.** The total expression level of core histone H2A, H2B and H3 was measured by western immunoblotting in strains. pRS315-WT refers to the strain that carries one copy of plasmid contains histone hta1 & htb1 genes inserted in pRS315 but deletes the original two H2A and H2B genes. **b-c.** Lethal test of the mutant with multiple interaction disrupted by plasmid shuffling. The results show that these mutations have a significant impact on cell viability. **d.** Principal component analysis (PCA) result compared the difference of transcription between WT and mutant strains **e.** Box plots of the enrichment of different histone post-tranlational modifications for all and up-regulated genes in R119G mutant strain. **f.** Volcano plots depict the gene expression changes of H2B-K111AH112A and H2B-R119E comparing to WT. **g.** Venn diagram showing the overlap the down-regulated and up-regulated genes for different mutans. **h.** GO enrichment analysis showed the function of the common up-regulated genes. **i.** HMR site silencing defects of different mutants.

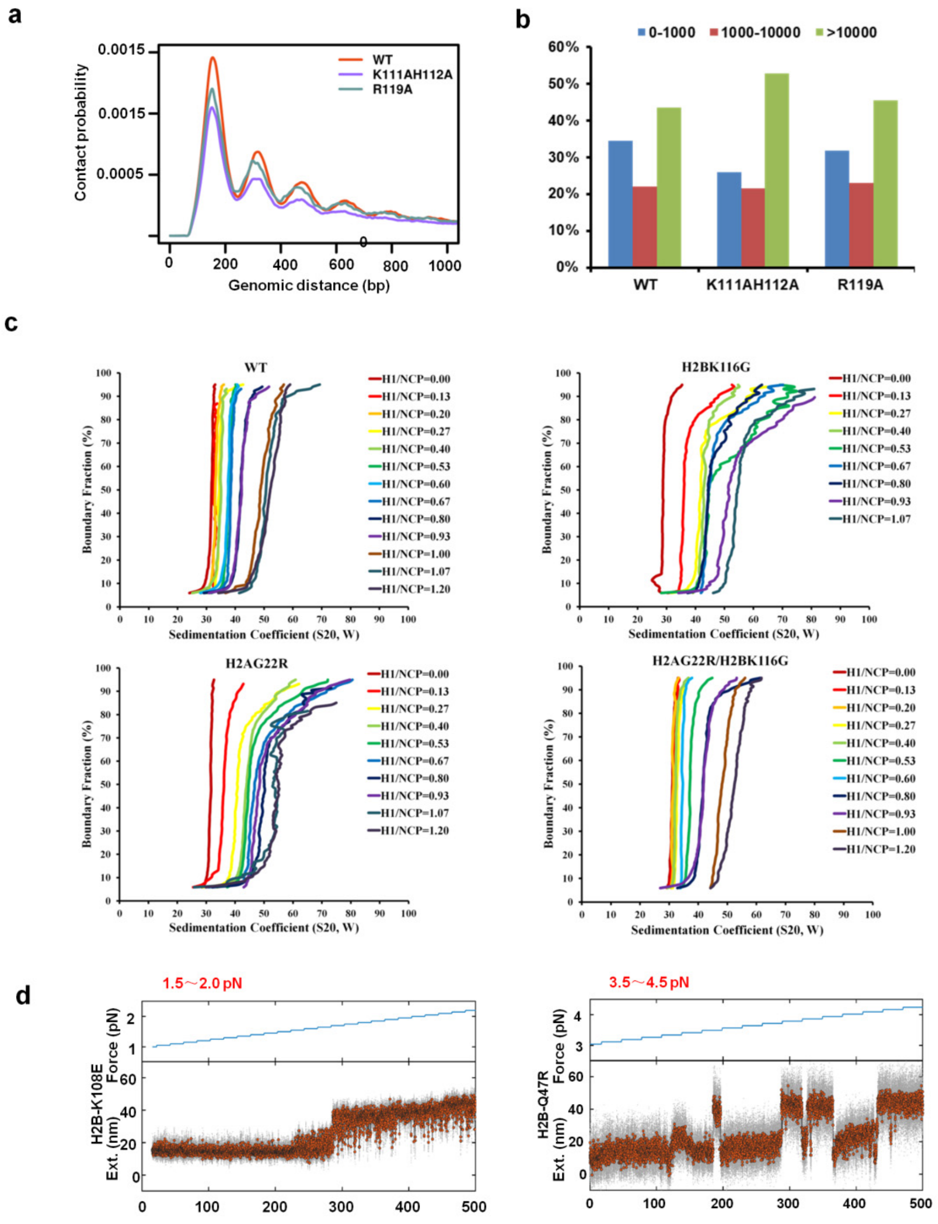

Supplementary information, Fig. S10 Assembly pattern and dynamic changes due to the histone mutations. a. Interaction frequency versus distance for yeast strains carrying either a single copy of H2A-H2B, or carrying the indicated histone mutants. Shown here are IN-OUT and OUT-IN read pairs only. **b.** Changes of interaction frequency with different distance shown as fig 2b in WT and two mutants in a. **c.** The sedimentation coefficient distributions for WT and different mutants with different H1 ratio using 12 × 177 bp nucleosome array. **d.** The unfolding trajectories of tetranucleosome reconstituted with mutant histones of H2B-K108E and H2B-Q47R using 4 × 177 bp nucleosome array.

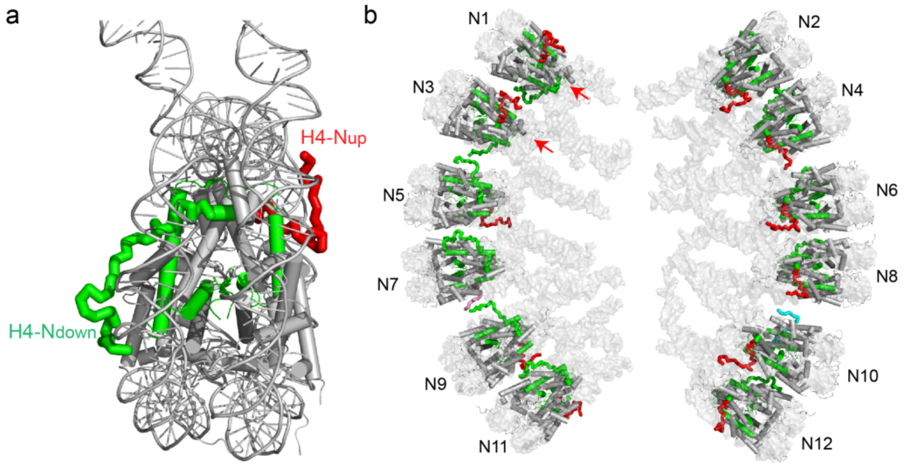

Supplementary information, Fig. S11 Structural asymmetries of the H4 N-tails in the H5-chromatin fiber. a. Two different structural conformations of the H4 N-tails (H4-Nup in red, H4-Ndown in green) in the chromatosome. All elements in the nucleosome are shown in grey except the two copies of H4 (green). **b.** The tandem arrangement of the H4-NTD in the chromatin fiber. H4 N-tails are shown as ribbon in different colors. Most of the H4 N-terminal tails display a unidirectionally tandem arrangement in the H5-chromatin fiber with some flexibilities, e.g., one of the H4-tails in N7 (pink) and N10 (cyan). N1 and N3 display an opposite direction as indicated by arrows.

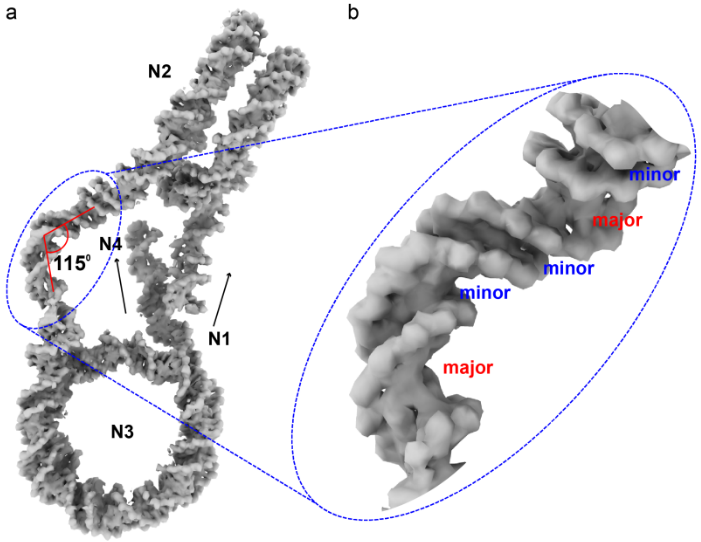

Supplementary information, Fig. S12 Torsional distortion of the linker DNA between N2 and N3 in the H5-chromatin fiber. a. The linker DNA between N2 and N3 shows a severe structural deformation with an approximately 115° kinking. **b.** An enlarged view of the kinked DNA. The major and minor grooves of DNA are indicated respectively.

Supplementary information, Table S1

Interactions within tetranucleosome units

Nucleosome stacks	Interaction regions	Residues involved in the interactions
N1:N3	^{1}H2B αC-^{3}H2A α2	^{1}K105-^{3}N68, ^{1}H106-^{3}E64, ^{1}K105-^{3}E64
	^{3}H2B αC-^{1}H2A α2	^{3}K105-^{1}E64
	^{1}H2B αC-^{3}H2B α1	^{1}T112-^{3}Q44,^{1}S109-^{3}V45
	^{1}H2B αC-^{3}H2A NTD	^{1}K117-^{3}G22,
	^{3}H2B αC-^{1}H2A NTD	^{3}K113-^{1}G22,
N2:N4	^{2}H2B αC-^{4}H2A α2	^{2}K113-^{4}E61, ^{2}S109-^{4}E64
	^{4}H2B αC-^{2}H2A α2	^{4}K105-^{2}E64
	^{4}H2B αC-^{2}H2B α1	^{4}T112-^{2}Q44,^{4}S109-^{2}Q44
	^{4}H2B αC-^{2}H2A NTD	^{4}K117-^{2}S19, ^{4}K117-^{2}L23, ^{4}K117-^{2}K15, ^{4}S120-^{2}K15
	^{2}H2B αC-^{4}H2A NTD	^{2}K117-^{4}G22
N5:N7	^{5}H2B αC-^{7}H2A α2	^{5}K105-^{7}E64
	^{7}H2B αC-^{5}H2A α2	^{7}K105-^{5}E64, ^{7}H106-^{5}E64
	^{5}H2B αC:^{7}H2B α1	^{5}T112-^{7}Q44
	^{5}H2B αC-^{7}H2A NTD	^{5}K117-^{7}G22
N6:N8	^{6}H2B αC-^{8}H2A α2	^{6}K105-^{8}N68
	^{8}H2B αC-^{6}H2A α2	^{8}K105-^{6}E64
	^{8}H2B αC-^{6}H2A NTD	^{8}K117-^{6}G22
N9:N11	^{9}H2B αC-^{11}H2A α2	^{9}K105-^{11}N68, ^{9}K105-^{11}E64
	^{11}H2B αC-^{9}H2A α2	^{11}K105-^{9}E64
	^{11}H2B αC-^{9}H2B α1	^{11}T112-^{9}Q44
	^{9}H2B αC-^{11}H2A NTD	^{9}K117-^{11}G22
	^{11}H2B αC-^{9}H2A NTD	^{11}K117-^{9}L23
N10:N12	^{12}H2B αC-^{10}H2A α2	^{12}K105-^{10}E64
	^{12}H2B αC-^{10}H2A NTD	^{12}S120-^{10}K15

Supplementary information, Table S2

Yeast strain used in this study

Strain	Genotype
YPH500	*MATα ade2–101, his3Δ200, leu2Δ1, lys2–801, trp1Δ63, ura3–52*
YPH499	*MATa ade2–101, his3Δ200, leu2Δ1, lys2–801, trp1Δ63, ura3–52*
HJ001	YPH500 *hta2-htb2Δ::TRP1*
HJ002	YPH500 *hta2-htb2Δ::TRP1/ CEN* pRS316-HTA1-HTB1
HJ003	YPH500 *hta2-htb2Δ::TRP1 hta1-htb1Δ::HIS3/ CEN* pRS316-HTA1-HTB1
HJ004	YPH500 *hta2-htb2Δ::TRP1 hta1-htb1Δ::HIS3/ CEN* pRS316-HTA1-HTB1 *CEN* pRS315-HTA1-HTB1
HJ005	YPH500 *hta2-htb2Δ::TRP1 hta1-htb1Δ::HIS3/ CEN* pRS315-HTA1-HTB1
HJ006	YPH500 *HMR A1::URA3*
HJ007	YPH500 *HMR A1::URA3 hta2-htb2Δ::TRP1 hta1-htb1Δ::HIS3/ CEN* pRS315-HTA1-HTB1
HJ008	YPH500 *HMR A1::ADE2 hta2-htb2Δ::TRP1 hta1-htb1Δ::HIS3/ CEN* pRS315-HTA1-HTB1
HJ009	YPH500 *HMR A1::URA3-600s hta2-htb2Δ::TRP1 hta1-htb1Δ::HIS3/ CEN* pRS315-HTA1-HTB1
HJ0010	YPH500 *hta2-htb2Δ::TRP1 hta1-htb1Δ::HIS3/ CEN hht2-hhf2Δ::HYG hht1-hhf1Δ::KAN/ CEN* pRS315-HTA1-HTB1-HHT1-HHF1

Supplementary information, Table S3

Phenotypic Analysis of budding yeast with H2A-H2B mutations

Strain	Viability	temprature stress	DNA damage	Lethality
H2A-G23E	ne	+++	+++	
H2A-G23R	ne	++	+++	
H2B-R119A	+++	+	+	
H2B-R119G	++	—	—	
H2B-R119E	++	—	—	
H2B-R119D	++	—	+	
H2B-R119H	+++	+	+	
H2B-R119K	ne	ne	ne	
H2A-G23R & H2B-R119A	ne	+++	+++	
H2A-G23R & H2B-R119G	ne	+++	+++	
H2A-G23K & H2B-R119A	ne	+++	+++	
H2A-G23K & H2B-R119G	ne	+++	+++	
H2A-G23R & H2B-R119E	+++	+	++	
H2A-G23E & H2B-R119E	+	—	—	
H2A-G23H & H2B-R119D	+++	+	+	
H2A-G23D & H2B-R119H	+++	++	++	
H2B-Q50A	ne	+++	+++	
H2B-Q50E	ne	++	++	
H2B-Q50R	ne	++	++	
H2B-Q50F	ne	+++	ne	
H2B-Q50W	ne	+++	ne	
H2B-S115E	ne	+++	++	
H2B-S115R	ne	+	+++	
H2B-T118E	ne	+	++	
H2B-T118R	ne	+	++	
H2B-T122E	ne	++	+++	
H2B-T122R	ne	+++	+++	
H2B-S115AT118A	ne	+++	+++	

H2B-S115ET118E	+++	—	+	
H2B-S115RT118R	ne	—	+	
H2B-S115AT118AT122A	+++	+	++	
H2B-K111A	+++	+	++	
H2B-H112A	+++	+	++	
H2B-K111AH112A	++	—	—	
H2B-K111E	++	—	+	
H2B-H112E	++	—	+	
H2B-K111EH112E	nt	nt	nt	Yes
H2B-K111AH112AR119A	nt	nt	nt	Yes
H2B-Q50AK111AH112A	++	—	—	
H2B-Q50AR119A	++	+	—	
H2B-Q50AR119G	nt	nt	nt	Yes
H2A-G23R & H2B-Q50AR119A	+++	++	+++	
H2A-G23R & H2B-Q50AR119G	+++	++	+++	
H2A-E62A	nt	nt	nt	Yes
H2A-E62D	++	+	+	
H2A-E65A	++	—	—	
H2A-E65G	nt	nt	nt	Yes
H2A-E65D	+++	++	+++	
H2A-R18A	+++	+	++	
H2A-R18P	++	+	+	
H2A-R18E	+	—	—	
H2A-K21A	+++	+	+	
H2A-L24A	ne	++	+++	
H2A-T25A	+++	+	+	
H2A-T25E	+++	+	+	
H2A-T25R	+++	++	+++	
H2B-Δ(126~130)	+++	+	++	
H2B-(126~129)4A	ne	+++	ne	
H2B-S126RS127R	ne	+	+++	
H2B-S126ES127E	ne	—	+	
H2B-Δ(123-130)	+	—	—	
H2B-K123A	+++	++	++	
H2B-K123A & H2A-G23K	ne	++	+++	

All the viability test were examined with spot tests on YPD plates or plates with different media and compared with WT. ne, no distinct effect; nt, didn't test; +++ >80%; ++ 30%–80%; + <30%; — almost no growth.

Supplementary information, Table S4

Data collection and refinement statistics

	H5-chromatin fiber	H5-NCP	GH5-NCP
	Data collection and processing		
Magnification	47,000	130,000	130,000
Voltage (kV)	300	300	300
Electron exposure ($e^-/Å^{-2}$)	60	60	60
Volta phase plate	No	Yes	No
Defocus range (μm)	-2.0 - -3.0	-0.5 - -1.0	-1.5 - -2.0
Pixel size (Å)	1.76	1.04	1.04
Symmetry imposed	C1	C1	C1
Initial particle images (no.)	43,402	536,264	276,401
Final particle images (no.)	13,670	33,753	45,705
Map resolution (Å)	3.6	3.3	3.6
FSC threshold	0.143	0.143	0.143
Refinement			
Initial model used (PDB code)	1KX5,1HST	- -	- -
Model resolution (Å)	3.5	- -	- -
FSC threshold	0.143	- -	- -
Model composition			
R.m.s. deviations			
Bond lengths (Å)	0.005	- -	- -
Bond angles (°)	0.817	- -	- -
Validation			
MolProbity score	1.99	- -	- -
Clashscore	12.34	- -	- -
Poor rotamers (%)	0.22	- -	- -
Ramachandran plot			
Favored (%)	94.33	- -	- -
Allowed (%)	5.62	- -	- -
Disallowed (%)	0.05	- -	- -
Model-to-data			
CC_{mask}	0.71	- -	- -
CC_{box}	0.75	- -	- -
CC_{peaks}	0.68	- -	- -
CC_{volume}	0.76	- -	- -

Supplementary information, Video legends

Video S1. The 3.6-Å resolution cryo-EM map of H5-chromatin fiber twisted by three tetranucleosomal units with straight linker DNA, and the 3D visualization of the N7 chromatosome density map.

Video S2. Cryo-EM map and 3D organization of linker histone H5 in H5-chromatin fiber.

Video S3. 3D visualization of chromatosome structure of H5-chromatin fiber, N7, presented in Fig. 1d.
